# Semimethylation is a feature of diffuse large B-cell lymphoma, and subgroups with poor prognosis are characterized by global hypomethylation and short telomere length

**DOI:** 10.1186/s13148-024-01680-4

**Published:** 2024-05-21

**Authors:** Olivia Carlund, Elina Thörn, Pia Osterman, Maja Fors, Andy Dernstedt, Mattias N. E. Forsell, Martin Erlanson, Mattias Landfors, Sofie Degerman, Magnus Hultdin

**Affiliations:** 1 Department of Medical Biosciences, Pathology, Umeå University, Umeå, Sweden; 2 Department of Clinical Microbiology, Infection and Immunology, Umeå University, Umeå, Sweden; 3 Department of Diagnostics and Intervention, Oncology, Umeå University, Umeå, Sweden

**Keywords:** Diffuse large-B cell lymphoma, High-grade B-cell lymphoma, Primary CNS lymphomas, DNA methylation, Telomere length, Predictive markers, Survival

## Abstract

**Background:**

Large B-cell lymphoma (LBCL) is the most common lymphoma and is known to be a biologically heterogeneous disease regarding genetic, phenotypic, and clinical features. Although the prognosis is good, one-third has a primary refractory or relapsing disease which underscores the importance of developing predictive biological markers capable of identifying high- and low-risk patients. DNA methylation (DNAm) and telomere maintenance alterations are hallmarks of cancer and aging. Both these alterations may contribute to the heterogeneity of the disease, and potentially influence the prognosis of LBCL.

**Results:**

We studied the DNAm profiles (Infinium MethylationEPIC BeadChip) and relative telomere lengths (RTL) with qPCR of 93 LBCL cases: Diffuse large B-cell lymphoma not otherwise specified (DLBCL, *n* = 66), High-grade B-cell lymphoma (*n* = 7), Primary CNS lymphoma (*n* = 8), and transformation of indolent B-cell lymphoma (*n* = 12). There was a substantial methylation heterogeneity in DLBCL and other LBCL entities compared to normal cells and other B-cell neoplasms. LBCL cases had a particularly aberrant semimethylated pattern (0.15 ≤ *β* ≤ 0.8) with large intertumor variation and overall low hypermethylation (*β* > 0.8). DNAm patterns could not be used to distinguish between germinal center B-cell-like (GC) and non-GC DLBCL cases. In cases treated with R-CHOP-like regimens, a high percentage of global hypomethylation (*β* < 0.15) was in multivariable analysis associated with worse disease-specific survival (DSS) (HR 6.920, 95% CI 1.499–31.943) and progression-free survival (PFS) (HR 4.923, 95% CI 1.286–18.849) in DLBCL and with worse DSS (HR 5.147, 95% CI 1.239–21.388) in LBCL. These cases with a high percentage of global hypomethylation also had a higher degree of CpG island methylation, including islands in promoter-associated regions, than the cases with less hypomethylation. Additionally, telomere length was heterogenous in LBCL, with a subset of the DLBCL-GC cases accounting for the longest RTL. Short RTL was independently associated with worse DSS (HR 6.011, 95% CI 1.319–27.397) and PFS (HR 4.689, 95% CI 1.102–19.963) in LBCL treated with R-CHOP-like regimens.

**Conclusion:**

We hypothesize that subclones with high global hypomethylation and hypermethylated CpG islands could have advantages in tumor progression, e.g. by inactivating tumor suppressor genes or promoting treatment resistance. Our findings suggest that cases with high global hypomethylation and thus poor prognosis could be candidates for alternative treatment regimens including hypomethylating drugs.

**Supplementary Information:**

The online version contains supplementary material available at 10.1186/s13148-024-01680-4.

## Background

Large B-cell lymphoma (LBCL) is the most common type of lymphoma with an incidence of 150,000 cases per year worldwide [1]. Diffuse large B-cell lymphoma not otherwise specified (DLBCL) accounts for more than 80% of LBCL cases and can arise either de novo or through transformation from an indolent lymphoma. DLBCL is an aggressive type of lymphoma with varying prognosis and treatment outcomes depending on the cell of origin (COO) [1, 2]. The introduction of high-intensive cytotoxic treatments and anti-CD20 monoclonal antibodies, as well as newer relapse treatments with chimeric antigen receptor T-cells and bispecific antibodies, has improved survival in DLBCL substantially in the last decades [1–4]. At present, two out of three patients are cured by the primary treatment, while the remaining third has a primary refractory or relapsing disease. Treatments may, however, result in both short- and long-term side effects that can affect overall survival negatively. Therefore, good predictive markers are needed to identify high- vs low-risk patients to better distinguish patients that need intensive treatment regimens and a milder treatment approach, respectively. Furthermore, there is a need for alternative treatment targets.

Diagnostics and subtyping of LBCL are based on morphology, immunophenotype, genetic aberrations, and clinical characteristics. COO in DLBCL is used as a survival predictor and is determined by gene expression profiling (activated B-cell-like (ABC), germinal center B-cell-like (GC), or unclassified) and/or immunohistochemistry (GC or nonGC) [5]. However, using only immunohistochemistry for subtyping has shown conflicting results regarding survival [1]. Furthermore, combined rearrangement of the *MYC*, *BCL2,* and/or *BCL6* genes (High-grade B-cell lymphoma (HGBL) with “double- or triple-hits”) is associated with worse survival and are recommended intensified treatment [6, 7]. Still, the risk stratification and subgrouping at diagnosis need further improvement [2, 6, 7].

Epigenetic alterations have been shown to regulate the replicative capacity of cells [8, 9]. Age-associated changes in DNA methylation (DNAm) on specific cytosine-phosphate-guanine (CpG) sites correlate well with chronological age, a phenomenon denoted epigenetic aging [10]. Accelerated epigenetic aging has been associated with all-cause mortality later in life, as well as with physical and cognitive fitness including longitudinal memory outcome and cancer [10–13]. Changed CpG site methylation profiles have been associated with the development and progression of cancer [14]. Kulis et al., showed that B-cells undergo global loss of methylation throughout maturation, with the most pronounced methylation changes occurring during late maturation stages [15]. B-cell neoplasms partially share methylation profiles with the maturation stage of their COO [16] and CpGs with dynamic methylation changes throughout B-cell maturation were highly affected in B-cell neoplasms [15]. In primary CNS lymphomas (PCNSL), an entity of LBCL that arises in the CNS, a specific DNAm profile has been described, separating it from DLBCL [17]. Several studies have investigated methylation differences between the DLBCL of GC and nonGC subtypes. The results are conflicting, with findings of both overlapping and distinct DNAm profiles between GC and nonGC subtypes [16, 18–21]. In DLBCL, DNAm heterogeneity in promoter regions has been associated with disease aggressiveness and worse progression-free survival [20, 22]. We have previously shown that Acute lymphoblastic leukemia (ALL) can be divided into two subgroups based on CpG island Methylator Phenotype (CIMP) classification, with different prognoses and different epigenetic ages [23, 24]. However, CIMP classification has not been evaluated in LBCL.

Along with epigenetic alterations, telomere attrition has been shown to regulate the replicative capacity of cells [8, 9]. Telomeres consist of a repetitive DNA sequence (TTAGGG) at the ends of the chromosomes and protect the chromosome ends from end-to-end fusions. At each replication, the telomeres are shortened as DNA polymerase is unable to fully copy the end of the telomere. When telomeres reach a certain length in cells in vitro, the cells stop dividing and enter a senescent state [25]. Telomere length (TL) in blood has been identified as a prognostic marker in various malignancies, and mutations in telomere-related genes are associated with shortened telomeres and increased risk for hematological malignancies [26, 27]. Recently, it has been shown that methylation changes at specific CpG sites are associated with TL [28, 29]. In this study, we analyzed global DNAm and TL in LBCL. Furthermore, we addressed their potential role in stratification of patient groups with LBCL and as predictive markers.

## Materials and methods

### Study population

The study population consisted of 96 adult patients diagnosed with large B-cell lymphoma between 2005 and 2018 at the University Hospital in Umeå, Sweden. The time interval for inclusion was chosen due to the introduction of the anti-CD20 antibody rituximab in 2005. Treatment guidelines for DLBCL remained mostly unchanged between 2005 and 2018. Cases with fresh frozen tumor tissue samples were selected. Sixty-nine cases of de novo DLBCL not otherwise specified, 7 HGBL, 8 PCNSL, and 12 transformations from indolent lymphomas (t-DLBCL) were included. Subtyping of DLBCL into GC or nonGC was performed according to the Hans algorithm based on immunohistochemical and/or flow cytometric expression of MUM-1, CD10, and BCL-6 [30]. All samples were analyzed with fluorescence in situ hybridization (FISH) retrospectively and were classified as HGBL if they had rearrangement of *MYC*, *BCL2,* and/or *BCL6*. All samples were microscopically analyzed regarding tumor cell content (i.e., tumor cell content < 20%, 20–39%, 40–59%, 60–79%, or 80–100%).

Clinical data was retrieved from medical records and from the national lymphoma register (INCA). The collected clinical data were: date of diagnosis, age, sex, lymphoma stage (according to Ann Arbor), lactate dehydrogenase level (LDH), performance status (according to WHO), treatment, response rate (complete remission, partial remission, or progressive disease), relapse rate, date of death (if applicable) and time for follow-up. The treatment regimens were divided into groups based on the choice of oncological treatment and whether the treatment was completed or not. Regimens including rituximab, cyclophosphamide, doxorubicin, vincristine, prednisone, and in some cases etoposide, that were given and completed with curative intent, were defined as R-CHOP-like treatment and only patients that received R-CHOP-like treatment were included in the survival analyses. Patients treated with other regimens (i.e., CNS treatment, other intensive treatment, radiotherapy, or only part of the planned treatment) were excluded from the survival analyses.

The study was approved by the Regional ethical review board in Umeå (Dnr 2016/258-31 and 2016/53-31) and in Uppsala (Dnr 2014/233), Sweden, and the patients/controls provided informed consent.

### Isolation of GC B-cells from tonsils

Normal GC-cells from tonsil (one sample) were collected at the Ear-Nose-Throat clinic at the University Hospital in Umeå, Sweden. A single-cell suspension of the tonsil was prepared as previously described [31]*.* Before sorting, the cells were thawed and washed in sterile PBS supplemented with 2% FBS, then stained with Zombie aqua (1:1000) (BioLegend, San Diego, CA), anti-human IgD-BV421 (clone IA6-2, BD Biosciences, Franklin Lakes, NJ), CD20-FITC (clone 2H7, BioLegend), CD38-PE (clone HIT2, BioLegend), and CD19-PE-CF594 (clone HIB19, BD Biosciences) for 30 min at RT. Cell sorting was performed on a BD FACSMelody (BD Biosciences) and viable GC B-cells were defined as CD19 + CD20 + CD38 + IgD-, with the sort rate < 1500 events/second. A total of 864,000 cells were sorted into RPMI-1640 medium (Gibco, Thermo Fisher Scientific, Waltham, MA), supplemented with 1% penicillin–streptomycin, 20 mM HEPES, and 50% FBS.

### Genomic DNA extraction of LBCL and normal GC B-cells

Genomic DNA was extracted using the AllPrep DNA/RNA mini kit (#80204, Qiagen, Hilden, GER) according to the manufacturer’s instruction with minor modifications: the addition of a four min incubation time to the washing steps, an additional washing step with buffer AW2, and five minutes incubation time to the DNA elution step. DNA concentration and quality were measured on a DeNovix DS-11 (DeNovix, Wilmington, DE). DNA integrity number (DIN) was calculated for each LBCL DNA sample using the Agilent 2200 TapeStation System and the Agilent Genomic DNA ScreenTape assay (Agilent Technologies, Santa Clara, CA) according to the manufacturer’s instructions.

### Relative telomere length measurements

Relative telomere length (RTL) was measured by qPCR using the method described by Cawthon [32] with minor modifications [33, 34]. Each DNA sample was measured in triplicates in separate telomere (*T*) and single-copy gene (*S*) reactions in a 384-well format. A reference curve from a cell line (CCRF-CEM) was included in every run to monitor the efficiency of the PCR. The difference between the cycle threshold (CT) for *T* and *S* was calculated for each sample to generate a *T*/*S* (2^−ΔCT^) value. The RTL values were obtained by dividing the *T*/*S* value for each sample by the *T*/*S* value from CCRF-CEM. All DNA samples were analyzed in two separate runs. The mean RTL values of the two runs were adjusted for age by including RTL values from an independent control cohort (DNA from peripheral blood leukocytes from 174 individuals, age 0–84 years) [34]. A local regression model (LOESS) was fitted to the RTL values of the control cohort to obtain the residuals. Then a LOESS was fitted to the squared residuals~age to obtain the estimated local standard deviation of controls. The standardized residuals of RTL (RTL_sres_) were obtained by dividing the residuals of LBCL with the local standard deviations of the control cohort.

### DNA methylation analysis

DNA (750–1000 ng) from the LBCL samples (*n* = 96) and the normal GC B-cells (*n* = 1) were bisulfite-converted with the EZ DNA Methylation Kit (Zymo Research, Irvine, CA) according to the manufacturer’s instructions. Efficient bisulfite conversion was confirmed by Methylight *ALU* PCR with the ALU-C4 primer/probe set [35]. The Infinium MethylationEPIC BeadChip (Illumina, San Diego, CA) with coverage of > 850,000 CpG sites was used for genome-wide assessment of DNA methylation profiles. The methylation levels for each CpG were given as *β*-values where 0 indicated that all copies of the CpG in a given sample were unmethylated and 1 indicated that they were methylated. The Genome Studio software V2011.1 (Illumina) and the R statistical software, version 4.2.2. (R core team) were used for pre-processing. The methylation data was imported into R using the *Minfi* package [36] and normalized with the background normalization package *Noob* [37]. Adjustments for the two different probe designs used by the EPIC array were performed with *BMIQ* [38]. Omitted sites during data pre-processing were CpGs located on the X and Y chromosomes (to avoid gender-related biases), CpGs with a detection *p* value > 0.05, CpGs located ≤ 5 bp from a known SNP, non-specific probes aligning to multiple loci, CpGs located in methylation quantitative trait locus (mQTL), and duplicated CpGs mapping to the same gene and position (Additional file 1: Figure S1) [39–41]. Publicly available DNAm data of normal B-cells from peripheral blood (*n* = 28) were downloaded from the Gene Expression Omnibus repository (GSE103541), normalized, and pre-processed according to our protocol and used as controls.

### Epigenetic clock models

The epigenetic age of LBCL cases was estimated using nine different clock models: Hannum’s clock [42], Horvath’s pan-tissue clock [10], the PhenoAge clock [43], epiTOC [13], MiAge [44], epiCMIT, epiCMIT-hyper, epiCMIT-hypo [16], and DNAmTL [29]. The models are based on specific CpG sites whose DNAm pattern alters over time. The output of Hannum’s clock, Horvath’s pan-tissue clock, and the PhenoAge clock are “biological years”, i.e. the physical and physiological condition of the cells. The output of the epiTOC and epiCMIT clocks is the mean *β*-value of the CpGs included in the respective model, which represents the accumulative increase in methylation at these locations over time. The output of the MiAge clock is a quantitative estimate of the total number of cell divisions. Lastly, DNAmTL estimates the length of telomeres and is stated as kilobases.

### CpG island methylator phenotype

The CIMP panel was originally based on 1347 CpG sites present on the Illumina Infinium 27 k Human Methylation Beadchip (Illumina) with a standard deviation of ≥ 0.3 within pediatric T-cell acute lymphoblastic leukemia (T-ALL) samples [45]. Of those CpGs, 1099 are present on the MethylationEPIC BeadChip, 1098 of these overlapped with the LBCL data set, and 1091 overlapped with the combined LBCL, normal GC B-cell, and normal B-cell data sets after our filtration and normalization. A sample was defined as CIMP- if ≤ 25% of the CpG sites in the panel had a *β* > 0.4, otherwise they were classified as CIMP+ [24].

### Statistical analysis

The R statistical software, version 4.2.2., was used for statistical analysis. Categorical variables were analyzed by Mann–Whitney *U* test, Kruskal–Wallis test followed by ad hoc test with Bonferroni correction, Pearson’s chi-squared test, and Fisher’s exact test. Continuous variables were analyzed with linear modeling. The ComplexHeatmap package was used for creating heatmaps [46] and the FactoMineR package was used for principal component analysis (PCA) [47]. The GeneGO MetaCore™ software (Thomson Reuters, New York, NY) was used for gene ontology analysis. Differentially methylated genes were analyzed using the enrichment analysis workflow tool and visualized in R. A previously published DNAm-based classifier of different subtypes of B-cell neoplasms was used to classify DLBCL cases into GC or nonGC subtypes [16]. The methylation variability score (MVS) is described in detail by Chambwe et al. [20]. The method requires Δ*β*-values of both cases and controls, where the Δ*β* of controls represents the expected normal methylation variation in a population. Here, the mean CpG *β*-values of the normal B-cells were used as baseline and the Δ*β*-values between the normal B-cells and the normal GC B-cells represented normal activation-specific methylation alterations. Briefly, the methylation difference (Δ*β*) between the normal B-cells and (1) the normal GC B-cells and (2) each LBCL sample was calculated. The Δ*β* density of each sample was obtained using a kernel density estimation with bandwidth = 0.01 and 1024 equally spaced points, ranging from − 1 to 1. The MVS was defined as the difference in area under the curve between the density function of a given LBCL sample and the normal GC B-cells and was calculated using the trapezoidal rule with *n* = 1023 subintervals. An MVS = 0 indicated no methylation differences between the given LBCL case and the normal GC B-cells.

Disease-specific survival (DSS) and progression-free survival (PFS) were analyzed in DLBCL (*n* = 56) and LBCL (*n* = 68) cases treated with R-CHOP-like regimens. DSS was measured from the day of diagnosis until death caused by lymphoma or until the last follow-up date (March 2022). PFS was measured from the day of diagnosis until progression, relapse, or last follow-up date. The median follow-up time was analyzed with reverse Kaplan–Meier plots. The factors evaluated were age, age-adjusted International Prognostic Index (aaIPI), entity, RTL_sres_, percentage of hypo-, semi-, and hypermethylated CpGs, mean *β*-value, global MVS, promoter MVS, epiTOC age, and CIMP classes. Quartile classification (mean *β*-value and percentage of hypo-, semi-, and hypermethylated CpGs) and median classification (age, MVS, and epiTOC) were based on the entire LBCL cohort (*n* = 93). RTL_sres_ were divided into three groups where cases with RTL_sres_ < − 3 were considered short, cases between − 3 and 3 were considered normal, and cases > 3 were considered long. aaIPI was stated as 0–1 and 2–3 since there were no events with aaIPI = 1 for the DLBCL-GC/nonGC cases. Kaplan–Meier curves were used to visualize univariable parameters and the Cox proportional hazards model was used for univariable and multivariable analysis. The log-rank test and Wald’s test were used to compare DSS and PFS between groups.

## Results

### The large B-cell lymphoma cases

Ninety-six LBCL cases were initially included in the study. Of those, three samples had a tumor cell content of less than 40% and were excluded. The remaining 93 samples were divided into subtypes: DLBCL with GC subtype (*n* = 36), DLBCL with nonGC subtype (*n* = 30), HGBL (*n* = 7), PCNSL (*n* = 8), and t-DLBCL (*n* = 12). The median age in the study cohort was 69 years (range 25–89 years) and the majority were men (59.1%, *n* = 55). R-CHOP-like treatment was given to 73.1% (*n* = 68) of the patients (Table 1).

**Table 1:** Clinical characteristics of the 93 LBCL cases

	All (*n* = 93)	DLBCL-GC (*n* = 36)	DLBCL-nonGC (*n* = 30)	HGBL (*n* = 7)	PCNSL (*n* = 8)	t-DLBCL (*n* = 12)
Age, median years (range)	69.0 (25–89)	65.5 (26–88)	69.0 (25–89)	70.0 (57–89)	66.0 (52–80)	70.0 (41–83)
Sex, percent men, % (*n*)	59.1 (55)	66.7 (24)	53.3 (16)	71.4 (5)	37.5 (3)	58.3 (7)
Stage at diagnosis, % (*n*)						
I	16.1 (15)	22.2 (8)	16.7 (5)	14.3 (1)	0	8.3 (1)
II	21.5 (20)	22.2 (8)	26.7 (8)	14.3 (1)	0	25.0 (3)
III	20.4 (19)	25.0 (9)	13.3 (4)	28.6 (2)	0	33.3 (4)
IV	41.9 (39)	30.6 (11)	43.3 (13)	42.9 (3)	100 (8)	33.3 (4)
Increased LDH, % (*n*)	50.5 (47)	47.2 (17)	63.3 (19)	57.1 (4)	37.5 (3)	36.3 (4)
Missing data, *n*	3	0	2	0	0	1
Performance status, % (*n*)						
0	41.9 (39)	47.2 (17)	53.3 (16)	42.9 (3)	0 (0)	33.3 (3)
1	38.7 (36)	47.2 (17)	26.7 (8)	28.6 (2)	37.5 (3)	66.7 (6)
≥ 2	16.1 (15)	5.6 (2)	20.0 (6)	28.6 (2)	62.5 (5)	0
Missing data, *n*	3	0	0	0	0	3
aaIPI, % (*n*)						
0	21.5 (20)	36.1 (13)	13.3 (4)	14.3 (1)	0	16.7 (2)
1	34.4 (32)	25.0 (9)	36.7 (11)	42.8 (3)	37.5 (3)	50 (6)
2	32.3 (30)	33.3 (12)	33.3 (10)	28.6 (2)	37.5 (3)	25.0 (3)
3	8.6 (8)	5.6 (2)	10.0 (3)	14.3 (1)	25.0 (2)	0
Missing data, *n*	3	0	2	0	0	1
Treatment, % (*n*)						
R-CHOP-like^a^	73.1 (68)	86.1 (31)	83.3 (25)	71.4 (5)	0	58.3 (7)
+ CNS-prophylaxis^b^	4.3 (4)	5.6 (2)	3.3 (1)	0	0	8.3 (1)
CNS^c^	8.6 (8)	0	0	0	100 (8)	0
Other intensive treatment^d^	3.2 (3)	0	3.3 (1)	0	0	16.7 (2)
Other^e^	8.6 (8)	5.6 (2)	6.7 (2)	28.6 (2)	0	16.7 (2)
No treatment	1.1 (1)	0	3.3 (1)	0	0	0
Missing data, *n*	1	1	0	0	0	0

*Abbreviations*: DLBCL-GC = Germinal-center B-cell-like diffuse large B-cell lymphoma. DLBCL-nonGC = Non-Germinal-center B-cell-like diffuse large B-cell lymphoma. HGBL = High-grade B-cell lymphoma. PCNSL = Primary CNS lymphoma. t-DLBCL = transformations from indolent lymphomas. LDH = Lactate dehydrogenase. aaIPI = age-adjusted International Prognostic Index (score for stage III-IV, increased LDH, performance status 2–4)

^a^Regimens including rituximab, cyclophosphamide, doxorubicin, vincristine, prednisone, and in some cases etoposide, completed with curative intent

^b^Regimens including high-dose methotrexate and/or cytarabine in addition to R-CHOP-like treatment

^c^Regimens including rituximab, methotrexate, cytarabine, and thiotepa (i.e., MATRIX), or rituximab, methotrexate, procarbazine, and vincristine (i.e., R-MPV) or high-dose single-methotrexate or single-temozolomide

^d^Regimens including rituximab, cisplatin, and cytarabine (R-DHAP) or rituximab, ifosfamide, carboplatin, and etoposide (R-IKE)

^e^Regimens with palliative intent (e.g., low dose cyclophosphamide or radiotherapy), patients receiving treatment but where the exact regimens could not be identified from medical records, and patients receiving only part of the planned treatment

### CpG methylation in LBCL and in normal cells

**Fig. 1:**
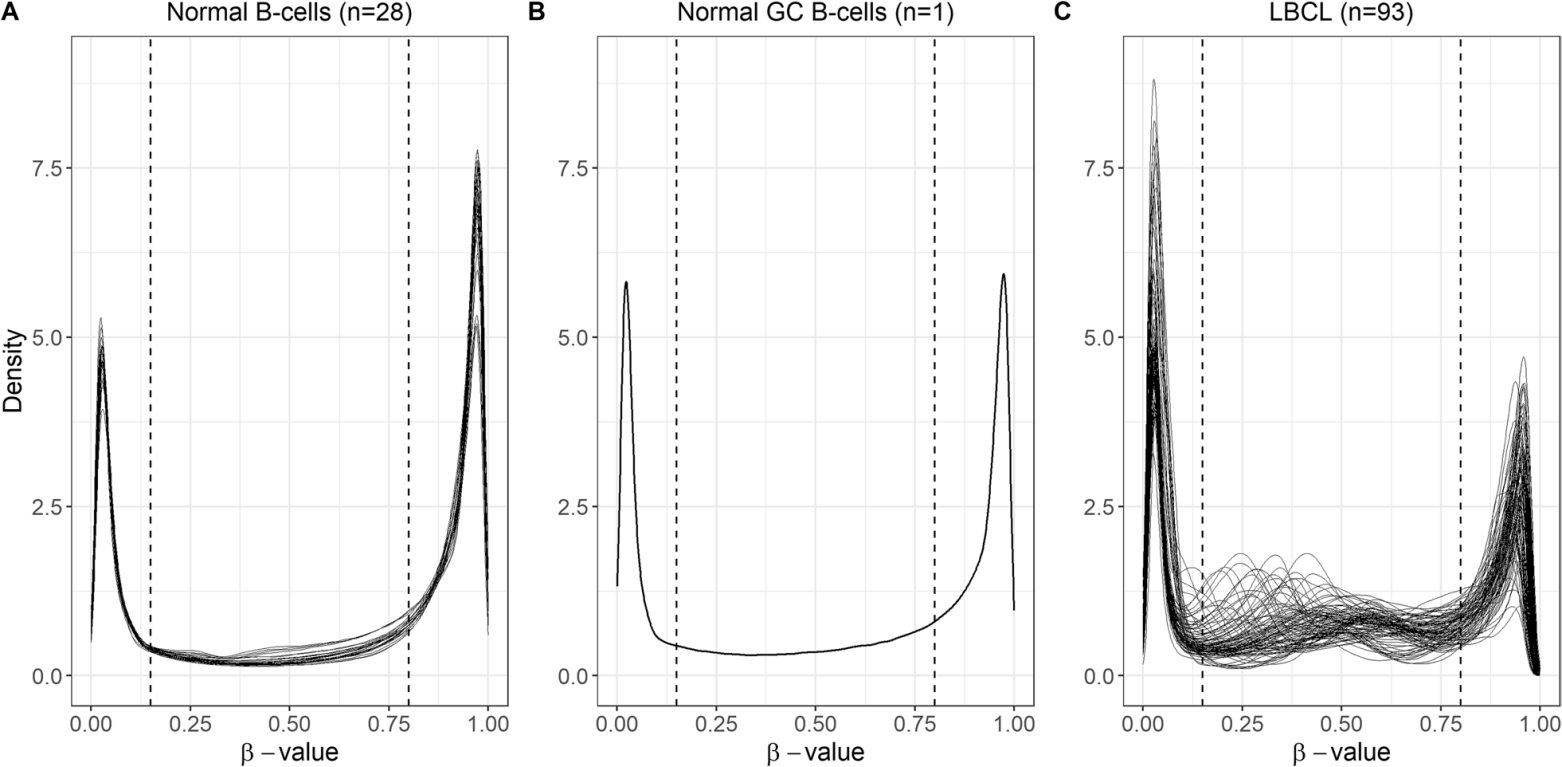
Methylation distribution in normal B-cells, normal GC B-cells, and LBCL. Density plots of *β*-value distribution for the 670,233 CpGs that overlapped between the three data sets. The vertical dashed lines mark the cutoff for 0.15 ≤ *β* ≤ 0.8. **A** Normal B-cells (*n* = 28), **B** normal GC B-cells (*n* = 1), **C** LBCL (*n* = 93)

The *β*-value distribution of the methylation arrays is expected to be bimodal with peaks around 0 and 1. In this study, we have called the peak around 0 the *hypomethylated CpGs* (*β* < 0.15), the peak around 1 the *hypermethylated CpGs* (*β* > 0.8), and the low-density region between the peaks the *semimethylated CpGs* (0.15 ≤ *β* ≤ 0.8). Semimethylated CpGs indicate that the copies of the CpG site are differentially methylated within a sample. Normal GC B-cells (*n* = 1) and normal peripheral B-cells (*n* = 28) were used as controls in the study. After quality control and filtration, 670,233 CpGs were overlapping between the LBCL (*n* = 93), the normal GC B-cells, and the normal B-cells data sets (Additional file 1: Figure S1). The *β*-values of the normal B-cells and the normal GC B-cells followed the expected distribution of the arrays (Fig. 1A and B). However, the LBCL cases had an aberrant methylation pattern with an increased number of semimethylated CpGs (Fig. 1C). This pattern was not entity-specific since similar patterns were seen in all LBCL subgroups (Additional file 1: Figure S2).

To evaluate if the heterogeneity observed in LBCL was present in other B-cell neoplasms, we downloaded methylation data from the Gene Expression Omnibus repository and normalized the data sets according to our protocol. The external data sets included B-cell precursor acute lymphoblastic leukemia (BCP-ALL, *n* = 663, GSE49031), Chronic lymphocytic leukemia (CLL, *n* = 75, GSE237299), Mantle cell lymphoma (MCL, *n* = 72, GSE237299), Multiple myeloma (Primary plasma cell leukemia, PCL, *n* = 14, GSE104770), DLBCL (*n* = 49, GSE237299 and GSE37362), and normal plasmablasts (*n* = 8, GSE72498) (Additional file 1: Figure S3). The BCP-ALL, CLL, MCL, and PCL methylation patterns were homogenous, although occasional heterogeneity was observed in MCL and PCL. The external DLBCL cases came from two separate studies and they both had heterogeneous methylation patterns, resembling our LBCL cases. These results suggest that aberrant semimethylation is a characteristic of DLBCL and other LBCL entities.

For each LBCL case, normal GC B-cells, and normal B-cells, the percentage of CpG sites classified as hypo-, semi-, and hypermethylated were calculated and compared (Additional file 1: Table S1). There was a significant difference in median CpG percentage between the three groups (*χ*^2^ = 19.696, *df* = 4, *p* < 0.001). In LBCL, the percentage of semimethylated CpGs was increased and the percentage of hypermethylated CpGs was decreased. For hypomethylated sites, the median percentage was slightly lower in LBCL, however, the range was larger compared to normal B-cells. The decreased percentage of hypermethylated sites and increased percentage of semimethylated sites in LBCL compared to normal cells were indicative of a gradual global loss of methylation.

### Principal component analysis of methylation variation

**Fig. 2:**
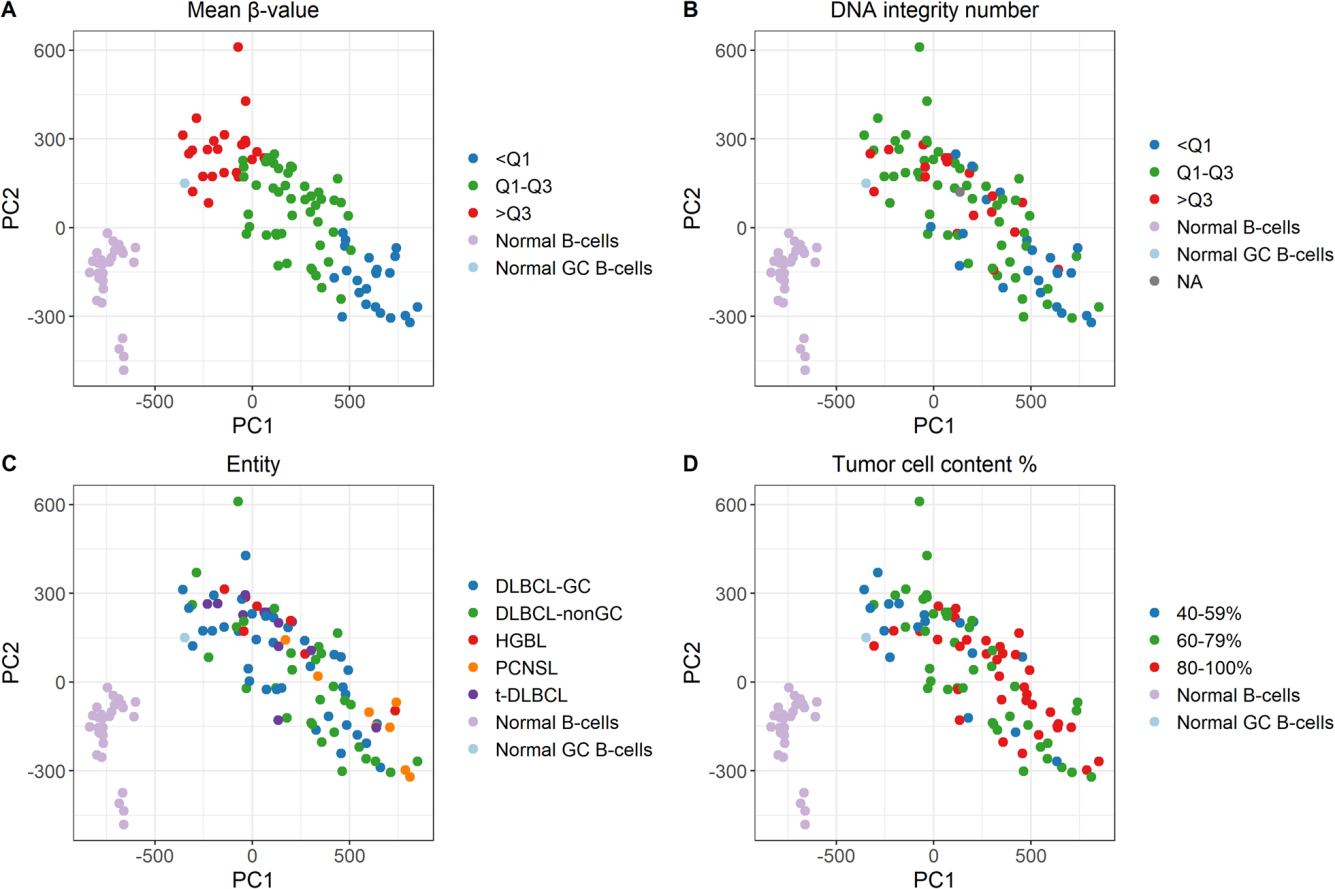
Principal component analysis of the LBCL cases, normal GC B-cells, and normal B-cells. The data set included 670,233 CpGs. **A** Mean β-value of each sample divided into quartiles, **B** DNA integrity number (DIN) divided into quartiles, **C** entity, **D** tumor cell content in percent

The first two principal components (PCs) of the genome-wide methylation data separated the data set into two clusters: one with the LBCL cases and the normal GC B-cells and one with the normal B-cells (Fig. 2). Since the PCA was based on *β*-values, there was a strong correlation between the mean *β*-value of each LBCL case (i.e. the mean *β*-value of all 670,233 CpG sites per sample) and PC1/PC2. There was, however, no specific correlation to DNA integrity number (DIN), entity, or tumor cell content, indicating that these factors were not the underlying cause of the methylation variations. PC1 explained 35.4% of the variation while PC2 explained 6.1%. PC3 (3.9%), PC4 (3.2%), and PC5 (2.8%) could not separate the data into distinct clusters and no association to DIN, entity, or tumor cell content was observed.

The normal cells were excluded and the PCA was performed on the LBCL data set only. PC1 and PC2 explained 23.3% and 5.6% of the variation, respectively, and were both correlated to the mean *β*-value of each LBCL case. We extracted the 10,000 CpGs that contributed most to PC1. The vast majority (99.8%) of them were classified as semimethylated CpGs, based on the mean *β*-value of each CpG, while the remaining 0.2% were classified as hypomethylated CpGs.

### Methylation characteristics based on mean *β*-value of each CpG site

The CpG sites were again divided into hypo-, semi-, and hypermethylated sites but this time based on the mean *β*-value of each CpG, for cases and normal cells, respectively (Additional file 1: Table S2). In LBCL, 143,101 (21.35% of the total CpGs) were classified as hypomethylated CpGs. The number of semi-, and hypermethylated sites were 384,689 (57.40%) and 142,443 (21.25%). There was a significant difference in median percentage between LBCL and the control groups (*χ*^2^ = 36.46, *df* = 4, *p* < 0.001). Venn diagrams of the CpG sites in each methylation group showed that there were few unique hypo- and hypermethylated sites in LBCL compared to normal cells (2863 and 387, respectively, corresponding to 2% and 0.3% of the total number of hypo/hypermethylated sites in LBCL) (Additional file 1: Figure S4). The unique number of semimethylated sites in LBCL was 181,879 (47%).

The gene-, and CpG island (CGI) annotation distributions in the hypo-, semi-, and hypermethylated groups were compared between LBCL and the normal GC B-cells, since the cell of origin of LBCL are closer to the GC B-cell maturation step than to the normal B-cells. Although there was a significant difference in the number of CpGs in each methylation group between LBCL and the normal GC B-cells (*χ*^2^ = 20.351, *df* = 2, *p* < 0.001), there was no significant difference in annotation distribution between the two groups (*p* = 1, Fisher’s exact test) (Additional file 1: Tables S2, S3, Figure S5).

**Fig. 3:**
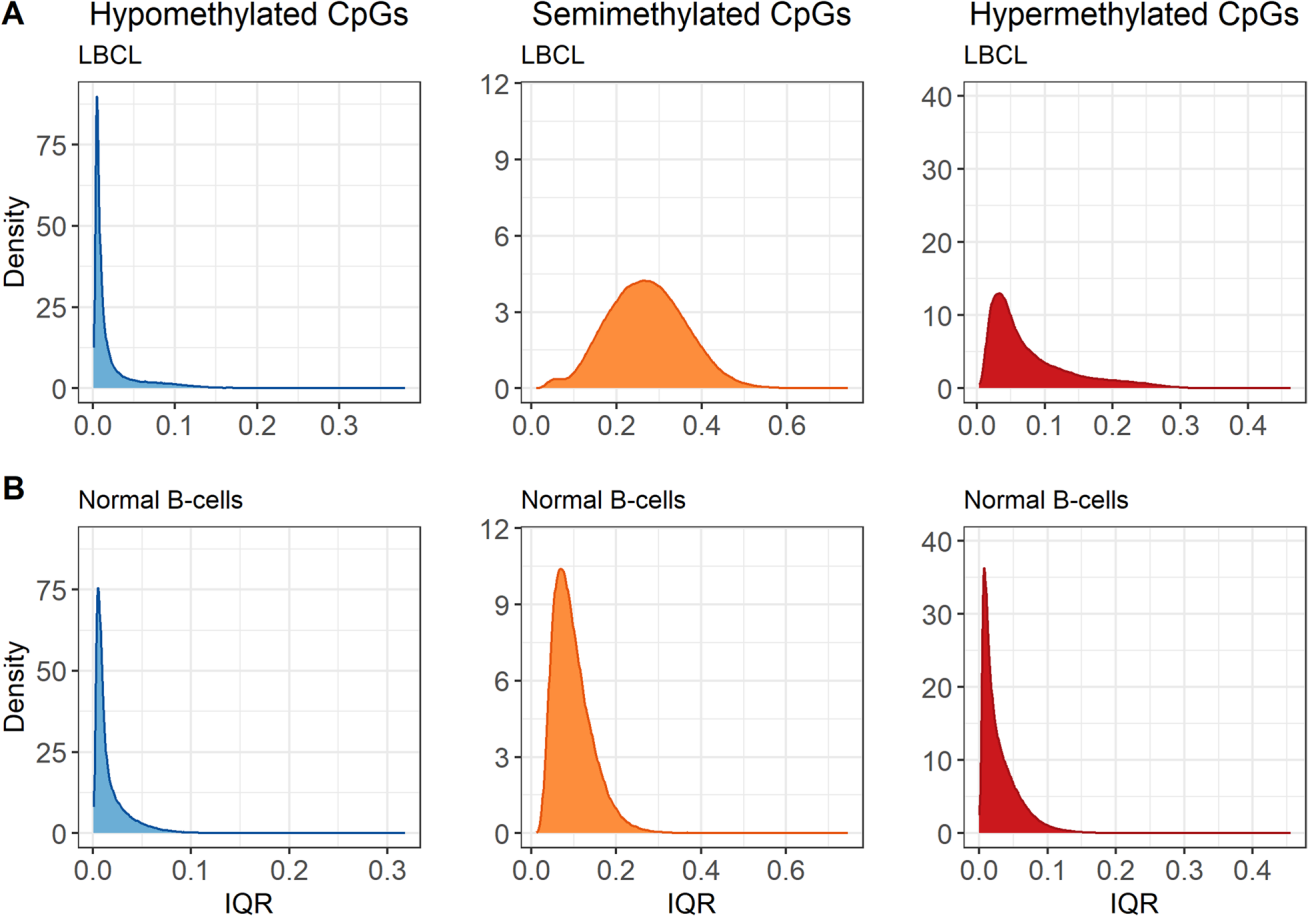
Interquartile range in LBCL cases and in normal B-cells. Density plots of IQR for the CpGs classified as hypo-, semi-, and hypermethylated based on the mean *β*-value of each CpG. **A** LBCL (*n* = 143,101, 384,689, and 142,443 CpGs) and **B** normal B-cells (*n* = 176,728, 140,086, and, 353,419 CpGs)

Since there was high methylation variability in LBCL when looking at individual cases (Fig. 1, Additional file 1: Table S1), interquartile ranges (IQR) were calculated for the hypo-, semi-, and hypermethylated CpGs. IQR could not be obtained from the normal GC B-cells due to the low sample size (*n* = 1). The CpGs classified as hypomethylated in LBCL (*n* = 143,101) and in the normal B-cells (*n* = 176,728) had low IQR, indicating low intertumor variability. However, the CpGs in the semi- and hypermethylated groups had larger variations in IQR, especially for LBCL (Fig. 3).

### Evaluation of entity-specific DNAm profiles in LBCL

The mean *β*-values and percentage of CpG sites in each methylation group (hypo-, semi-, and hyper) were calculated for each entity. PCNSL had a significantly lower mean *β*-value than DLBCL-GC (*p* = 0.037) and t-DLBCL (*p* = 0.014) and significantly increased percentage of hypomethylated CpGs compared to HGBL (*p* = 0.007) (Table 2). Additionally, t-DLBCL had a significant increase of hypermethylated CpGs compared to DLBCL-nonGC (*p* = 0.027). A previous study identified that PCNSL had significantly increased CGI methylation compared to DLBCL [17]. We filtered out all CpGs annotated as “Islands” (*n* = 133,550) and calculated the mean *β*-value of islands for each sample. Our results confirmed that PCNSL had a significant increase in CGI methylation compared to both DLBCL-GC (*p* = 0.018) and DLBCL-nonGC (*p* = 0.012) (Table 2).

**Table 2:** DNAm characteristics of LBCL entities

	DLBCL-GC (*n* = 36)	DLBCL-nonGC (*n* = 30)	HGBL (*n* = 7)	PCNSL (*n* = 8)	t-DLBCL (*n* = 12)	*p* value
Mean *β*-value	0.50 + 0.09	0.47 + 0.08	0.53 + 0.05	0.43 + 0.04	0.53 + 0.04	**0.005**^**a**^
Hypomethylated CpGs %	23.82 + 3.44	24.43 + 4.78	21.59 + 1.34	41.78 + 16.74	22.70 + 1.73	**0.011**^**b**^
Semimethylated CpGs %	45.17 + 14.34	49.06 + 8.37	47.21 + 3.87	29.68 + 21.12	42.45 + 6.01	0.051
Hypermethylated CpGs %	28.63 + 8.83	25.76 + 9.46	30.24 + 4.75	25.67 + 4.74	32.48 + 5.20	**0.014**^**c**^
Global CGI mean β-value	0.24 + 0.05	0.24 + 0.04	0.26 + 0.03	0.28 + 0.02	0.24 + 0.04	**0.014**^**d**^
Global MVS	0.71 + 0.55	0.80 + 0.52	0.58 + 0.20	0.89 + 0.15	0.47 + 0.31	**0.005**^**e**^
Promoter-associated MVS	0.48 + 0.30	0.50 + 0.21	0.63 + 0.18	0.58 + 0.10	0.40 + 0.18	0.072

^a^PCNSL vs DLBCL-GC *p* = 0.037, PCNSL vs t-DLBCL *p* = 0.014

^b^PCNSL vs HGBL *p* = 0.007

^c^t-DLBCL vs DLBCL-nonGC *p* = 0.027

^d^PCNSL vs DLBCL-GC *p* = 0.018, PCNSL vs DLBCL-nonGC *p* = 0.012

^e^t-DLBCL vs DLBCL-nonGC *p* = 0.037, t-DLBCL vs PCNSL *p* = 0.050

The statistical analysis was performed with the Kruskal–Wallis test (*p* value) followed by ad hoc test with Bonferroni correction (significant *p* values in footnotes). Significant *p* values (*p* < 0.05) are indicated as bold text. Median and IQR values are stated

**Fig. 4:**
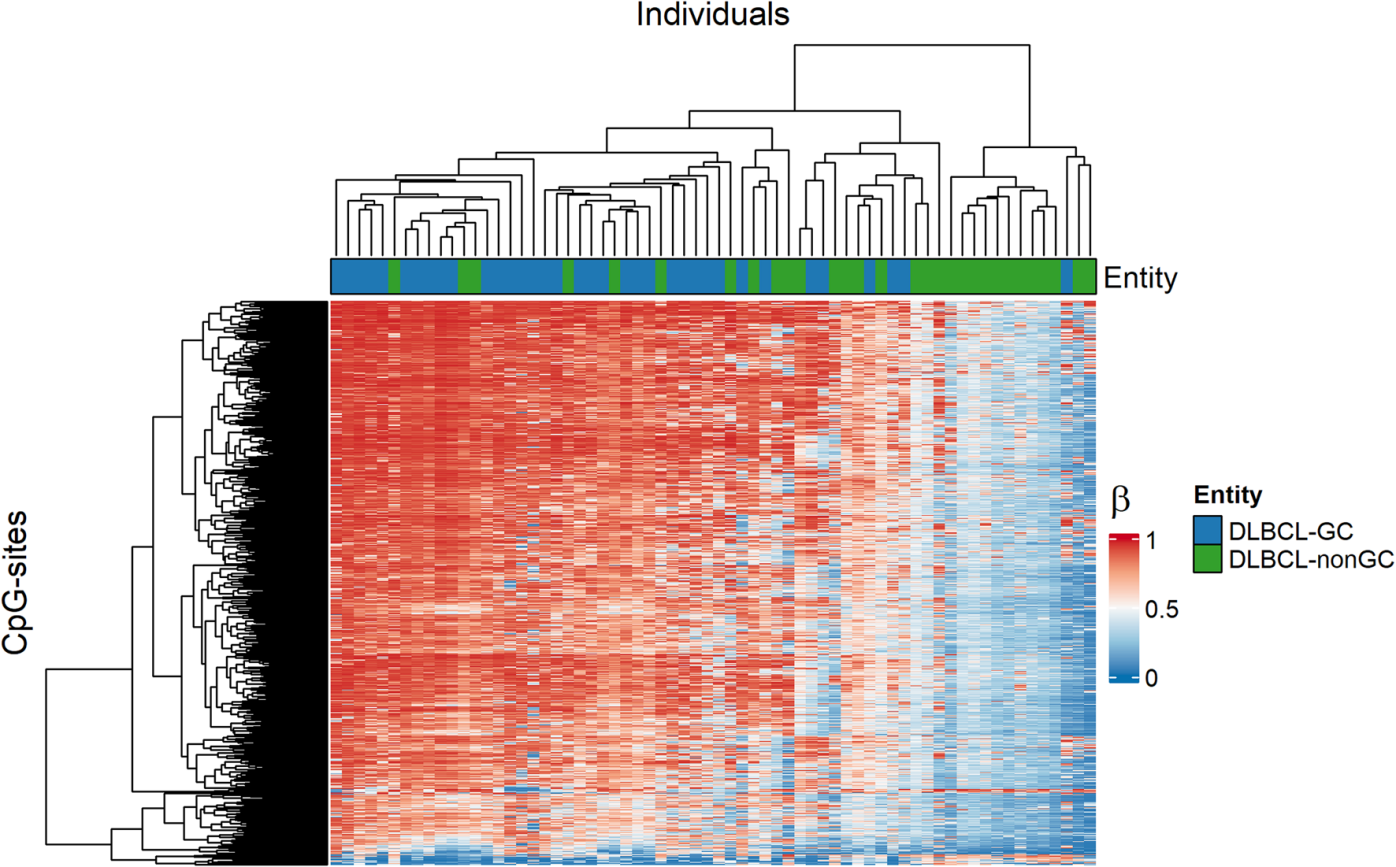
Heatmap clustering of the 1148 DM-CpGs identified between DLBCL-GC and DLBCL-nonGC. The cutoff was mean |Δ*β*|≥ 0.2. Rows correspond to CpGs, which are color-coded after methylation level: *β* = 0 is blue and *β* = 1 is red. Columns represent samples and the annotation bar below the column dendrogram is colored after entity

A cluster analysis was performed on differentially methylated CpGs (DM-CpGs) between DLBCL-GC and DLBCL-nonGC. The cutoff for a DM-CpG was set to mean |Δ*β*|≥ 0.2 and was based on Fig. 1, where the CpGs in the hypermethylated group had a *β* range of 0.2 (0.8–1). Cluster analysis of the 1148 DM-CpGs identified between DLBCL-GC/nonGC could not differentiate between the two entities (Fig. 4). A gene ontology analysis of the genes (*n* = 639) associated with these DM-CpGs did not identify any significant networks (Additional file 1: Figure S6). The threshold for DM-CpGs was increased to mean |Δ*β*|≥ 0.3 to increase the gap between the two groups, which resulted in only 4 sites. These results indicated there were few entity-specific methylation differences between DLBCL-GC and DLBCL-nonGC. The LBCL entities were further compared to the normal GC B-cells to identify unique DM-CpGs within each entity. The threshold was set to mean |Δ*β*|≥ 0.4 since the normal GC B-cells were non-malignant and large methylation differences were expected. In total, 3064 DM-CpGs were overlapping between all entities. In DLBCL-GC, 212 DM-CpGs did not overlap with any of the other entities and the corresponding number for DLBCL-nonGC was 902 (Additional file 1: Figure S7). However, clustering of the unique DM-CpGs from DLBCL-GC and DLBCL-nonGC in heatmaps could not separate the entities from each other, again indicating that DM-CpGs retrieved from mean methylation differences were not representative of the subgroups (Additional file 1: Figure S8).

In addition to mean methylation differences, a methylation variability score (MVS) adapted from Chambwe et al. [20] was calculated for each individual LBCL sample (Additional file 1: Figure S9). An MVS of 0 corresponded to no methylation difference between a given LBCL sample and the normal GC B-cells. There were minor differences between the entities. The t-DLBCL had decreased MVS compared to DLBCL-nonGC (*p* = 0.037) and to PCNSL (*p* = 0.050) but besides that, there was no significant difference in MVS between any subgroup (Table 2). Linear regression analysis of mean *β*-value and MVS for each case showed a negative correlation (*R*^2^_adj_ = 0.708, *p* < 0.001), indicating that cases with high mean methylation were more similar to normal GC B-cells than cases with low mean methylation, which confirmed the results of the PCA (Fig. 2A). MVS was also calculated for the CpGs located in promoter-associated regions: TSS1500/TSS200 (1500–200/200–0 bases upstream of the transcription start site), 5’UTR (within the 5’ untranslated region), and 1stExon (*n* = 217,835). However, after Bonferroni correction, there was no significant difference between any of the entities (Table 2). In conclusion, DLBCL showed an aberrant methylation pattern compared to the normal GC B-cells. They also had large intertumor heterogeneity, obstructing entity separation based on DNAm.

In contrast to our results, it has previously been suggested that DLBCL-GC and DLBCL-nonGC can be distinguished based on methylation profiles. A pan-B-cell neoplasm classifier was published in 2020, which could differentiate between ALL, MCL, CLL, DLBCL, and Multiple myeloma (MM) and then further classify the lymphomas into subgroups [16]. There were 66 DLBCL samples in our cohort and the pan-B-cell neoplasm classifier correctly classified 58 of those as DLBCL, giving it a sensitivity of 88%. For classification into DLBCL entities, the sensitivity and specificity were 42% and 87% for GC and 87% and 42% for nonGC. Thus, in our cohort, the classifier could identify DLBCL but was not applicable for GC/nonGC subdivision.

### Epigenetic age in LBCL

**Fig. 5:**
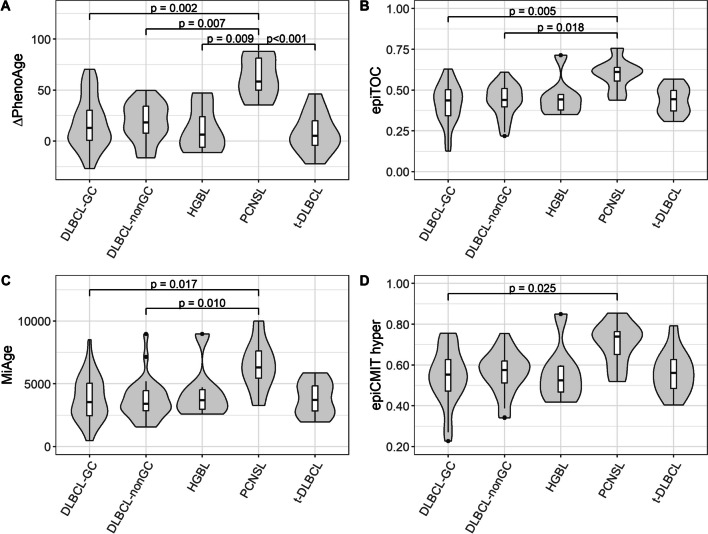
Violin plots of the epigenetic age in LBCL cases. The boxplots within each violin represent the spreading, with the IQR represented as the box and the median as the black vertical line within the box. Black dots within the violin plot represent outliers. The statistical analysis was performed with the Kruskal–Wallis test followed by ad hoc test with Bonferroni correction. **A** ΔPhenoAge, **B** epiTOC, **C** MiAge, **D** epiCMIT hyper

The epigenetic age in the LBCL entities was estimated using nine different DNAm clock models (Additional file 1: Table S4). The output of Hannum’s clock, Horvath’s pan-tissue clock, and the PhenoAge clock were years, hence the residuals (biological age – chronological age) were stated. The other clocks reflect mitosis and were not adjusted for chronological age. PCNSL had a significantly older epigenetic age than other entities using the PhenoAge, epiTOC, MiAge, and epiCMIT-hyper clocks (Fig. 5). Interestingly, the t-DLBCL did not have a significantly older epigenetic age compared to the DLBCL-GC/nonGC subtypes, even though they could be expected to have gone through more cell divisions since they had transformed from an indolent to an aggressive state.

### CpG island Methylator Phenotype (CIMP)

We have previously shown that CIMP classification was relevant in ALL, where the CIMP- cases had worse prognosis compared to the CIMP + cases [23, 24, 45]. The LBCL cases were classified as CIMP- (*n* = 18) if ≤ 25% of the CpG sites in the CIMP panel had a *β* > 0.4, otherwise they were classified as CIMP+ (*n* = 75). The distribution of CIMP- cases in each entity were: 28% (DLBCL-GC, *n* = 10), 13% (DLBCL-nonGC, *n* = 4), 29% (HGBL, *n* = 2), 0% (PCNSL, *n* = 0), and 17% (t-DLBCL, *n* = 2). Cluster analysis of the CIMP panel in a heatmap could not separate between entities, except for the PCNSL cases (Additional file 1: Figure S10). The CIMP panel was based on promoter-associated CGIs and these results indicated that promoter-associated CGI methylation was heterogenous in all LBCL subtypes, except in PCNSL.

### Telomere length dynamics in LBCL entities

**Fig. 6:**
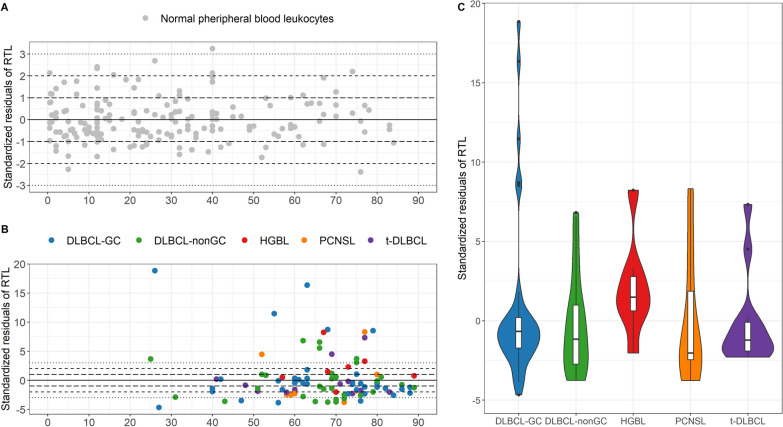
Standardized residuals of RTL in LBCL and normal blood leukocytes. RTL_sres_ were calculated based on peripheral blood leukocytes from 174 healthy individuals, 0–84 years. The solid line marks the age-adjusted mean value and the dashed lines represent residual ± 1, ± 2, and ± 3. **A** The normal peripheral blood leukocytes, **B** the LBCL cases, **C** Violin plots of the LBCL cases. The boxplots within each violin represent the spreading, with the IQR represented as the box and the median as the black vertical line within the box

The RTL of the LBCL cases were adjusted for age, here, stated as RTL_sres_. The control material used to adjust for age was DNA from peripheral blood leukocytes (*n* = 174, 0–84 years) and their RTL_sres_ range was − 2.4 to 3.3 [34]. LBCL on the other hand had an RTL_sres_ range of − 4.7 to 18.9 and the five samples with the longest RTL_sres_ were all DLBCL-GC (Fig. 6). Plotting the RTL_sres_ against entity showed that PCNSL had the lowest median value and largest IQR of all the entities and that the DLBCL-nonGC cases had a lower median value and larger IQR than the DLBCL-GC cases (Fig. 6C, Additional file 1: Table S5). However, there was no significant difference in RTL_sres_ between any of the entities (*p* = 0.306, Kruskal–Wallis test).

RTL_sres_ was not correlated to DIN (*R*^2^_adj_ = 0.016, *p* = 0.116) or tumor cell content (*p* = 0.256, Kruskal–Wallis test), indicating that neither the fragmentation of the DNA nor the tumor cell content influenced RTL_sres._ Further, the relationship between RTL_sres_ and methylation profiles was studied. There was no correlation to global MVS (*R*^2^_adj_ = 0, *p* = 0.334), mean *β*-value (*R*^2^_adj_ = − 0.010, *p* = 0.759), or percentage of hypo- (*R*^2^_adj_ = − 0.011, *p* = 0.914), semi- (*R*^2^_adj_ = − 0.007, *p* = 0.560), or hypermethylated CpGs (*R*^2^_adj_ = − 0.006, *p* = 0.489). Additionally, there was no correlation between the DNAmTL clock and RTL_sres_ (*R*^2^_adj_ = − 0.005, *p* = 0.467).

### Survival analysis

**Fig. 7:**
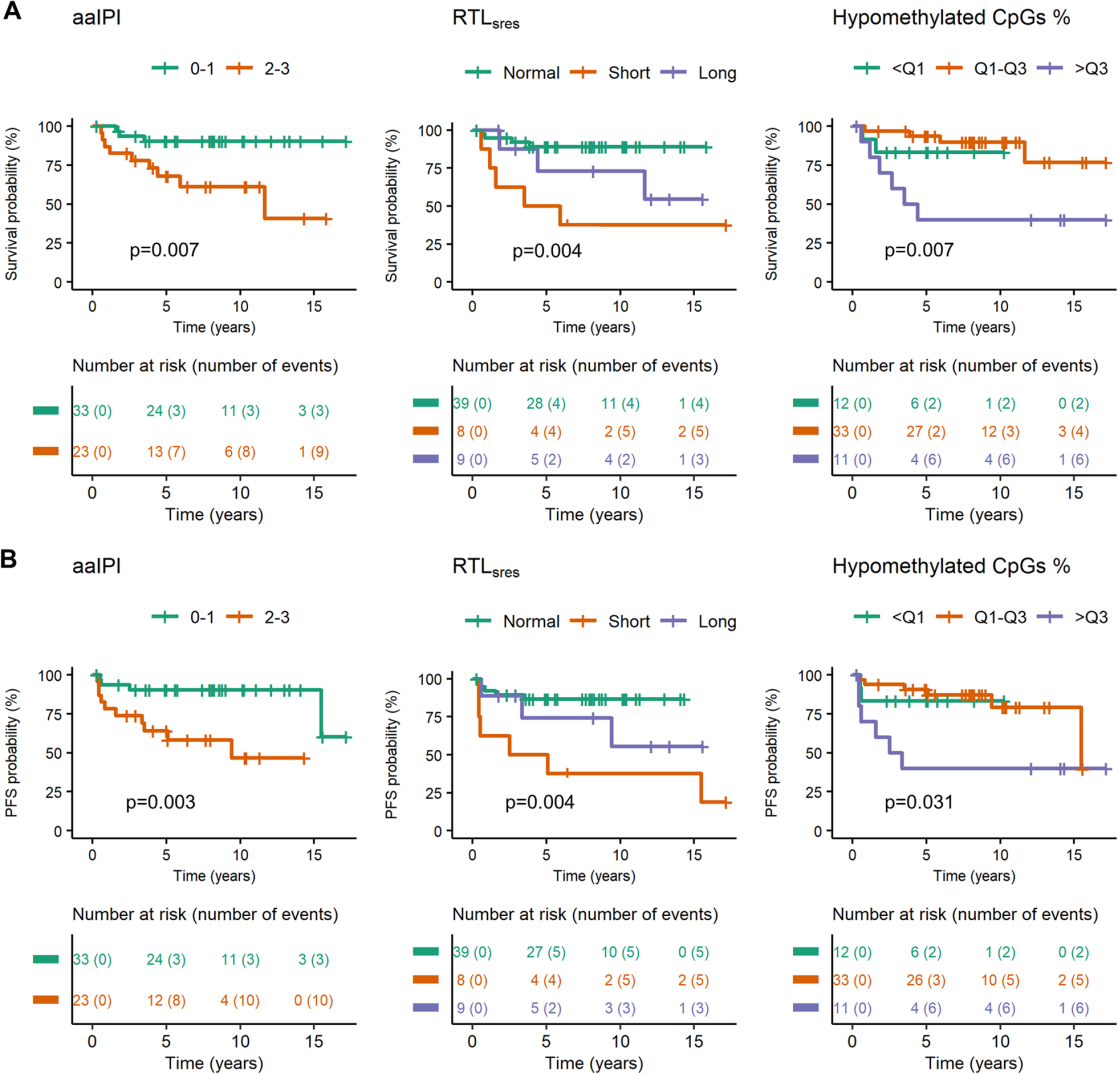
Disease-specific survival and progression-free survival analysis. Significant Kaplan–Meier curves and risk tables of DLBCL-GC and DLBCL-nonGC cases treated with R-CHOP-like regimens (*n* = 56). The cause of death was stated as death by lymphoma (*n* = 12). Progression was stated as progression or relapse at any time during follow-up (*n* = 14). *p* values were retrieved from the Log-rank test. **A** DSS, **B** PFS

DSS and PFS were analyzed in DLBCL-GC/nonGC cases treated with R-CHOP-like regimens (*n* = 56) and in all LBCL cases treated with R-CHOP-like regimens (*n* = 68). The factors included were age, aaIPI, entity, RTL_sres_, percentage of hypo-, semi, and hypermethylated CpGs, mean *β*-value, global MVS, promoter MVS, epiTOC age, and CIMP classes. The reference groups in the Cox proportional hazard models were decided based on the results from Kaplan–Meier plots (Fig. 7, Additional file 1: Figure S11, S12, S13).

For the DLBCL-GC/nonGC cases, 12 patients (21%) died from lymphoma and 14 patients (25%) had a relapse. The median follow-up time was 8 years for DSS and PFS. Factors associated with worse DSS and PFS in the Kaplan–Meier analysis were: aaIPI (DSS: 90% (< median), 41% (≥ median) and PFS: 60% (< median), 47% (≥ median)), RTL_sres_ (DSS: 89% (normal), 38% (short), 55% (long) and PFS: 87% (normal) 19% (short), 56% (long)), and hypomethylated CpGs (DSS: 83% (< Q1), 77% (Q1–Q3), 40% (> Q3) and PFS: 83% (< Q1), 40% (Q1–Q3), 40% (> Q3)) (Fig. 7). Univariable Cox proportional hazard analysis gave the same significant factors (Additional file 1: Table S6). Age and entity were not significant in the univariable analysis but were included in the multivariable analysis since they are known risk factors in DLBCL [1, 2]. The factors associated with worse prognosis in the multivariable analysis were aaIPI 2–3 (DSS: HR 6.366, 95% CI 1.558–26.024 and PFS: HR 6.866, 95% CI 1.746–27.003) short RTL_sres_ (DSS: HR 5.077, 95% CI 1.121–22.997), and hypomethylation > Q3 (DSS: HR 6.920, 95% CI 1.499–31.943 and PFS: HR 4.923, 95% CI 1.286–18.849) (Table 3).

**Table 3:** Multivariable survival analysis in DLBCL-GC and DLBCL-nonGC treated with R-CHOP-like regimens

Variables (*n* = 56)	Events (*n* = 12)	DSS		Events (*n* = 14)	PFS	
		HR (95% CI)	*p* value		HR (95% CI)	*p* value
Age						
< median (*n* = 31)	7	Reference		9	Reference	
≥ median (*n* = 25)	5	1.064 (0.278–4.068)	0.927	5	0.699 (0.199–2.456)	0.576
aaIPI						
0–1 (*n* = 33)	3	Reference		4	Reference	
2–3 (*n* = 23)	9	6.366 (1.558–26.024)	**0.010**	10	6.866 (1.746–27.003)	**0.006**
Entity						
GC (*n* = 31)	5	Reference		5	Reference	
nonGC (*n* = 25)	7	2.401 (0.566–10.185)	0.235	9	3.013 (0.815–11.135)	0.098
RTL_sres_						
Normal (*n* = 39)	4	Reference		5	Reference	
Short (*n* = 8)	5	5.077 (1.121–22.997)	**0.035**	6	3.585 (0.860–14.945)	0.080
Long (*n* = 9)	3	1.911 (0.358–10.192)	0.449	3	1.414 (0.297–6.736)	0.664
Hypomethylated CpGs %						
Q1–Q3 (*n* = 33)	4	Reference		6	Reference	
< Q1 (*n* = 12)	2	1.162 (0.196–6.887)	0.869	2	0.732 (0.132–4.060)	0.722
> Q3 (*n* = 11)	6	6.920 (1.499–31.943)	**0.013**	6	4.923 (1.286–18.849)	**0.020**

Multivariable Cox proportional hazard model of disease-specific survival (DSS) and progression-free survival (PFS) in DLBCL-GC and DLBCL-nonGC treated with R-CHOP-like regimens. Quartile- and median classification were based on the entire LBCL cohort. Significant *p* values (*p* < 0.05, Wald’s test) are indicated as bold text

For all LBCL cases treated with R-CHOP-like regimens, 16 patients (24%) died from lymphoma and 20 patients (29%) had a relapse. The median follow-up time was 8 years for DSS and PFS. Factors associated with worse DSS and PFS in the Kaplan–Meier analysis were: aaIPI 2–3 (DSS: 86% (< median), 38% (≥ median) PFS: 53% (< median), 44% (≥ median)), short RTL_sres_ (DSS: 83% (normal), 38% (short), 49% (long), PFS: 76% (normal), 19% (short), 50% (long)) and hypomethylation > Q3 (DSS: 58% (< Q1), 76% (Q1-Q3), 42% (> Q3), PFS: 39% (< Q1), 38% (Q1-Q3), 42% (> Q3)) (Additional file 1: Figure S12, Table S7). The factors associated with worse prognosis in the multivariable analysis were aaIPI 2–3 (DSS: HR 5.079, 95% CI 1.632–15.813 and PFS: HR 3.645, 95% CI 1.340–9.910), HGBL (DSS: HR 17.427, 95% CI 2.359–128.755 and PFS: HR 12.130, 95% CI 2.028–72.545), t-DLBCL (PFS: HR 6.190, 95% CI 1.171–32.710), short RTL_sres_ (DSS: HR 6.011, 95% CI 1.319–27.397 and PFS: HR 4.689, 95% CI 1.102–19.963), and hypomethylation > Q3 (DSS: HR 5.147, 95% CI 1.239–21.388) (Additional file 1: Table S8).

**Fig. 8:**
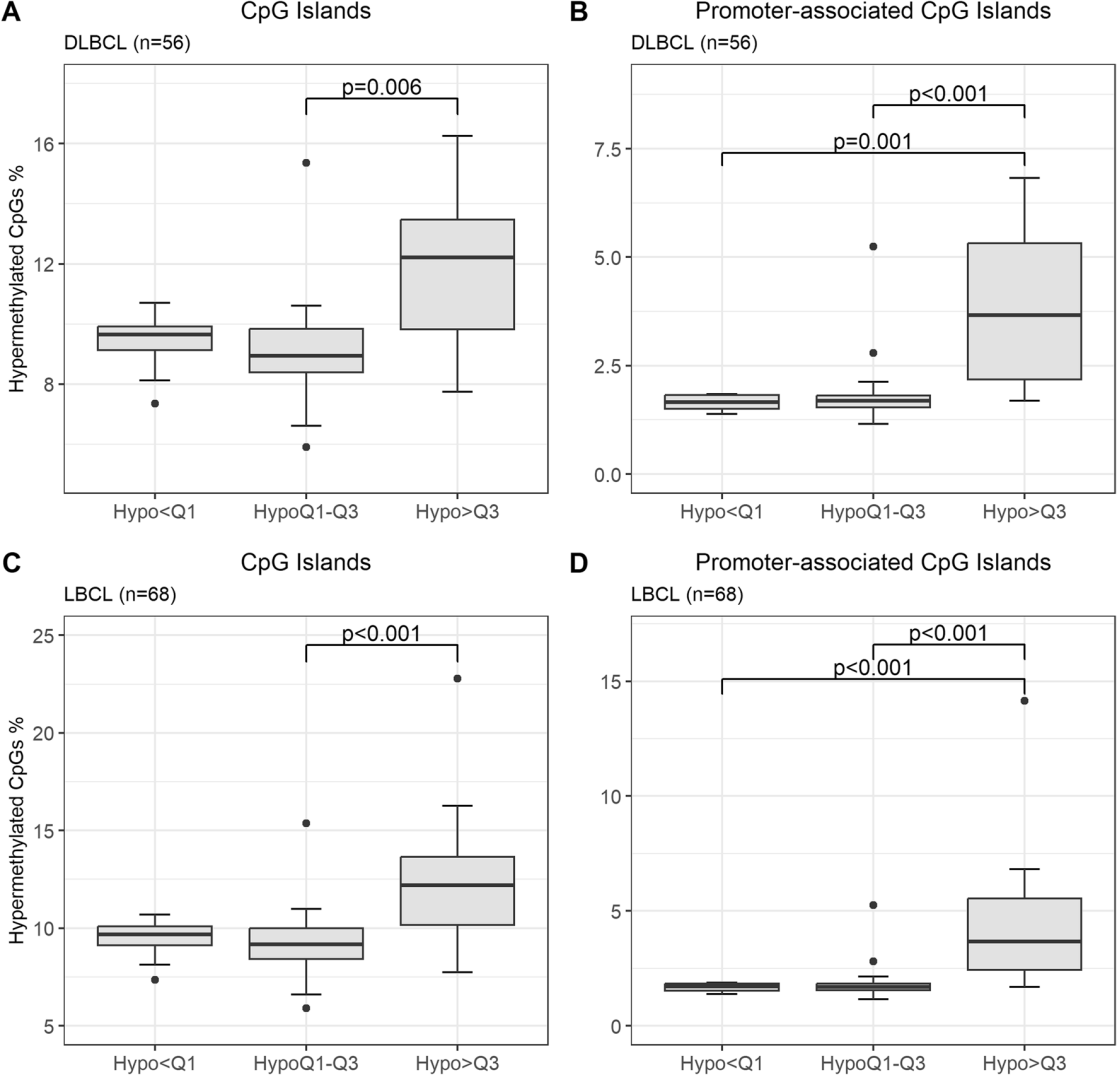
CGI hypermethylation in relation to global hypomethylation in cases treated with R-CHOP-like regimens. Boxplots showing the relationship between CGI hypermethylation (y-axis) and global hypomethylation (x-axis) in the cases treated with R-CHOP-like regimens. The quartile classification was based on the entire LBCL cohort. The statistical analysis was performed with the Kruskal–Wallis test followed by ad hoc test with Bonferroni correction. **A** Percentage of hypermethylated CGIs in DLBCL treated with R-CHOP-like regimens (*n* = 56). **B** Percentage of hypermethylated CGIs in promoter-associated regions (TSS1500/TSS200 (1500-200/200-0 bases upstream of the transcription start site), 5’UTR (within the 5’ untranslated region), and 1stExon) in DLBCL treated with R-CHOP like regimens (*n* = 56). **C** Percentage of hypermethylated CGIs in LBCL treated with R-CHOP like regimens (*n* = 68). **D** Percentage of hypermethylated CGIs in promoter-associated regions in LBCL treated with R-CHOP like regimens (*n* = 68)

DLBCL cases with a high frequency of global hypomethylation (Hypo > Q3) had poor DSS and PFS. Further analysis of these cases revealed that the Hypo > Q3 group had a significantly higher frequency of hypermethylated CGIs (I.e., percent of CGIs with a *β* > 0.8) compared to the HypoQ1-Q3 group (*p* = 0.006) (Fig. 8A). There was a trend toward higher frequency of hypermethylated CGIs in Hypo > Q3 compared to Hypo < Q1, although not significant (*p* = 0.051). Global hypomethylation and increased CGI methylation are common characteristics of malignant cells. Further, hypermethylation of CGIs in promoter regions is associated with silencing of tumor-suppressing genes [48]. We filtered out the promoter-associated CGIs (*n* = 86,250) and observed an even stronger association between the increased frequency of hypermethylated CGIs in Hypo > Q3 compared to HypoQ1–Q3 (*p* < 0.001) and Hypo < Q1 (*p* = 0.001) (Fig. 8B). Similar results were seen in LBCL (Fig. 8C and D). Importantly, PCNSL patients, which had increased CGI methylation compared to DLBCL-GC/nonGC, were not treated with R-CHOP-like regimens and thus they did not influence the results. Next, we examined which CGIs in the R-CHOP-like treated DLBCL cohort that had enriched CGI hypermethylation in the Hypo > Q3 group and the biological networks that were associated with these genes. The Hypo < Q1 and HypoQ1–Q3 samples were combined into one group (Hypo < Q1–Q3). We filtered out all CGIs that were hypermethylated in at least 70% of the Hypo > Q3 group and in no more than 30% of the Hypo < Q1–Q3 group, resulting in 550 CGIs located in 402 unique genes. Gene ontology analysis showed significant networks including signal transduction, reproduction, neurophysiological processes, development, cell adhesion, and cardiac development (Additional file 1: Figure S14A). Using the same criteria for the promoter-associated CGIs identified significant networks in signal transduction, neurophysiological processes, development, and cell adhesion (Additional file 1: Figure S14B).

## Discussion

LBCL is known to be a biologically heterogeneous disease regarding genetic, phenotypic, and clinical features. Several studies have confirmed the presence of epigenetic alterations, including DNAm, in LBCL, mainly focusing on promoter regions (around 50 K CpGs), selected genes relevant for tumorigenesis, or genome-wide DNAm using the 450 K arrays. Previous studies have also investigated DNAm changes between DLBCL subtypes with conflicting results [17, 19–21]. Therefore, we sought to clarify this by analysis of genome-wide DNAm (> 850,000 CpGs) in a large, well-characterized cohort of DLBCL, and compare with HGBL, PCNSL, and t-DLBCL. Additionally, TL has been shown to interact with epigenetic mechanisms and has been identified as a prognostic marker in cancer [49]. We aimed to elucidate whether DNAm and TL were of relevance for the subclassification and prognosis of LBCL.

The LBCL cases in our study had a heterogenous DNAm pattern with large inter- and intratumor heterogeneity. In comparison to normal cells, there was a large proportion of semimethylation in LBCL and the inter- and intratumor heterogeneity was high in CpGs classified as semi- and hypermethylated. In contrast, the hypomethylated CpGs had low intertumor variability, although there was a subset of cases with a very large proportion of hypomethylated CpGs. Global loss of methylation and increased semimethylation is a feature of normal B-cell maturation, most notably in late maturation stages, starting with the GC B-cells where massive reconfiguration of DNAm has been described [15]. Intratumor methylation heterogeneity in B-cell neoplasms has previously been identified in a subset of CLL cases [50] and in MM [51, 52] but not in BCP-ALL [53]. This raises the question of whether DNAm heterogeneity is a general feature of B-cell neoplasms derived from mature B-cell stages, possibly in GC-derived processes. We compared the LBCL cases to previously published methylation data and our results demonstrated that DNAm heterogeneity was more prominent in DLBCL and other LBCL entities than in BCP-ALL, CLL, MCL, and MM (PCL). Normal GC B-cells undergo intense DNA replication, somatic hypermutations, and class-switch recombination, accompanied by epigenetic alterations. Thus, the normal GC B-cells harbor various DNAm alterations and GC-derived B-cell neoplasms could be expected to have heterogenous DNAm, resulting in intertumor variation. Further, as LBCL originates from cells with active mechanisms for DNAm alterations, and mutations in epigenetic regulators are common in GC B-cell derived lymphomas [54], the tumors may retain or increase these properties rendering subclones of the tumor with an even more heterogeneous methylome.

We could not identify entity-specific DNAm profiles or gene ontology processes associated with DM-CpGs in DLBCL. Several studies have previously examined the methylomes of DLBCL-ABC/GC [16, 18–22]. Some studies have identified distinct methylation signatures in ABC and GC but due to the large DNAm heterogeneity in DLBCL, the sites constituting the basis for subgrouping might be cohort-dependent. The extensive remodeling of the DNA methylome within germinal centers may increase the DNAm heterogeneity among GC-cells and obstruct attempts to separate DLBCL subtypes originating from these cells solely on DNAm profiles.

Although we did not observe major differences in DLBCL entity-specific DNAm patterns, it is worth highlighting that PCNSL exhibited a distinctive profile. The PCNSL had significantly lower mean *β*-value compared to DLBCL-GC and t-DLBCL, and significantly increased methylation in CGIs compared to DLBCL-GC/nonGC. Global MVS was also significantly higher in PCNSL compared to t-DLBCL. These results were in line with a previous study that identified increased CGI methylation in PCNSL compared to DLBCL, however, they also reported that PCNSL differed more from normal lymph nodes than DLBCL, which we did not identify with respect to MVS [17]. Further, all PCNSL cases were CIMP+ and had increased epigenetic age compared to the other entities. The majority of the CpGs constituting the CIMP panel, the epiTOC- and the MiAge clocks are located in islands while the epiCMIT-hyper clock is derived from gene body and intergenic CpGs located at constitutive H3K27me3-containing regions, i.e. Polycomb repressed regions. Increased methylation in intergenic CGIs and in Polycomb target genes have previously been identified in PCNSL compared to normal cells [55]. However, whether these differences are a consequence of an increased mitotic rate or if they are lymphoma-specific requires further evaluation. As t-DLBCL originates from an indolent lymphoma that often has been present for many years, an increased epigenetic age could have been expected in this entity but our results did not support that hypothesis. This could indicate that the subclones giving rise to the aggressive lymphoma did not have the long replicative history that we anticipated. An alternative explanation could be that the methylation profile of the indolent lymphomas was rather stable during progression, as indicated by previous studies of Follicular lymphoma, where aberrant methylation was suggested to be an early event in lymphomagenesis [56, 57]. However, our cohort of t-DLBCL was small, and further studies are required to characterize DNAm changes and epigenetic aging during transformation.

Telomere maintenance is one of the hallmarks of cancer and malignant cells bypass telomere-mediated senescence by upregulating the telomere maintenance mechanisms, leading to immortalization [8]. TL varied both between and within tumor types in a study analyzing over 18,000 samples across 31 cancer types [58]. A study of B-cell leukemias/lymphomas showed rather similar median TL between entities, however, DLBCL-GC cases displayed large variation and a subset of cases with very long telomeres [59]. In our cohort, RTL_sres_ in LBCL were heterogenous, with the largest variation observed in DLBCL-GC. The reason why long RTL was observed in some DLBCL cases is unknown. Telomerase activity is the most common way to maintain telomeres in tumors, but 10–15% of solid tumors use Alternative Lengthening of Telomeres (ALT) for telomere maintenance. So far, ALT has not been described in hematological cells [60]. Long telomeres are associated to ALT phenotype and the long telomeres observed in DLBCL could be related to ALT phenotype but these results need further evaluation in the future.

Survival analysis of DLBCL-GC/nonGC cases treated with R-CHOP-like regimens showed that a high aaIPI score and a high percentage of hypomethylated CpGs were associated with both worse DSS and PFS in multivariable analysis. Since aaIPI scores are a well-established prognostic factor, that result was expected. However, global hypomethylation outperformed both age and entity as an independent prognostic factor, which is in line with a previous study [61]. The reason why DLBCL patients with a large proportion of hypomethylation have worse outcomes is unknown. The cases with a high percentage of hypomethylated CpGs had a higher frequency of hypermethylated CGIs compared to the cases with less hypomethylation, which could be relevant for promoter regions. Gene ontology analysis of the genes that had enriched promoter-associated CGI hypermethylation in the Hypo > Q3 group identified a few significant pathways, but the relevance of these pathways needs further evaluation. We did not have any data on gene expression or mutations in genes associated with epigenetics in our DLBCL cohort, but one hypothesis could be that subclones with a highly hypomethylated methylome, which correlated with hypermethylated CGIs, could have a survival advantage, e.g. by inactivating tumor suppressor genes or inducing resistance against treatment. Thus, the cases in this group might be candidates for alternative treatment regimens, including demethylating therapy, such as azacitidine and decitabine. We were not able to evaluate DSS and PFS in the DLBCL-GC and DLBCL-nonGC entities separately as the number of events in the quartiles was low, but that analysis could be of interest in future studies. The LBCL group was also difficult to interpret as some cases of HGBL and t-DLBCL were included. HGBL requires more intensified treatment [6, 7] and in multivariable analysis, HGBL had worse DDS and PFS. The included cases of t-DLBCL also had worse PFS in multivariable analysis which is in accordance with previous studies [62, 63]. The potential relevance of global hypomethylation for prognosis in HGBL and t-DLBCL needs confirmation in unrelated larger cohorts.

CIMP classification together with minimal residual disease is a strong prognostic marker in T-ALL and it also had prognostic relevance in relapsed BCP-ALL [24, 45]. We evaluated CIMP classification but it was neither of importance for subclassification of DLBCL/LBCL nor a significant prognostic marker. Thus, the CIMP panel, that was developed in ALL which originates from precursor cells, did not seem to be relevant in our cohort of mature LBCL.

Short RTL_sres_ were associated with worse DSS in multivariable analysis, but not with PFS in DLBCL-GC/nonGC cases. There are conflicting results regarding the impact of TL as a prognostic marker in cancer. A meta-analysis by Zhang and colleagues showed that short TL was associated with increased cancer mortality risk and cancer progression, especially in CLL [49]. A previous study of DLBCL could not see any prognostic impact of TL when dividing the cohort at the median length but didn´t show results for additional subgroups of TL [59]. In our cohort, the cases with the shortest TL had worse outcomes. Short telomeres can increase the risk for additional genetic aberrations and could be an explanation for these results [25].

In conclusion, our results showed that semimethylated, heterogeneous DNAm patterns were a feature of DLBCL but not common in other B-cell neoplasms. Furthermore, DLBCL cases with short RTL_sres_ and with a high percentage of hypomethylated CpGs were associated with worse DSS in multivariable analysis. These hypomethylated cases also had a higher frequency of hypermethylated CGIs and in this group of DLBCL patients, treatment with hypomethylating agents could potentially be beneficial but needs to be investigated in future studies.

### Supplementary Information

**Additional file 1**.

## Abbreviations

ABC: Activated B-cell-like
aaIPI: Age-adjusted International Prognostic Index
COO: Cell of origin
CGI: CpG island
CIMP: CpG island Methylator Phenotype
DM-CpG: Differentially methylated CpGs
DLBCL: Diffuse large B-cell lymphoma not otherwise specified
DSS: Disease-specific survival
DNAm: DNA methylation
GC: Germinal center B-cell-like
HGBL: High-grade B-cell lymphoma
LBCL: Large B-cell lymphoma
MVS: Methylation variability score
PCNSL: Primary CNS lymphoma
PFS: Progression-free survival
t-DLBCL: DLBCL transformed from indolent lymphoma
RTL_sres_: Standardized residuals of relative telomere length

## Declarations

### Availability of data and materials

The data sets generated during the current study are available at the Gene Expression Omnibus (GEO) repository, with the accession number (GSE255869).
