# Supplementary material for: Semimethylation is a feature of diffuse large B-cell lymphoma, and subgroups with poor prognosis are characterized by global hypomethylation and short telomere length
Source: Clin Epigenetics. 2024 May 21;16:68. doi: 10.1186/s13148-024-01680-4 (PMC11110316; doi:10.1186/s13148-024-01680-4)
Supplement: Supplementary file 1 — Additional file 1. [file 13148_2024_1680_MOESM1_ESM.docx]

# Additional file 1

Contents

[Supplementary table 1. 2](#_Toc158841110)

[Supplementary table 2. 3](#_Toc158841111)

[Supplementary table 3. 4](#_Toc158841112)

[Supplementary table 4. 5](#_Toc158841113)

[Supplementary table 5. 6](#_Toc158841114)

[Supplementary table 6. 7](#_Toc158841115)

[Supplementary table 7. 9](#_Toc158841116)

[Supplementary table 8. 11](#_Toc158841117)

[Supplementary figure 1. 12](#_Toc158841118)

[Supplementary figure 2. 13](#_Toc158841119)

[Supplementary figure 3. 14](#_Toc158841120)

[Supplementary figure 4. 15](#_Toc158841121)

[Supplementary figure 5. 16](#_Toc158841122)

[Supplementary figure 6. 17](#_Toc158841123)

[Supplementary figure 7. 18](#_Toc158841124)

[Supplementary figure 8. 19](#_Toc158841125)

[Supplementary figure 9. 20](#_Toc158841126)

[Supplementary figure 10. 22](#_Toc158841127)

[Supplementary figure 11. 23](#_Toc158841128)

[Supplementary figure 12. 25](#_Toc158841129)

Table S1.
Hypo-, semi-, and hypermethylated CpGs in LBCL cases, normal B-cells, and normal GC B-cells.

|  | **LBCL (n=93)** | **Normal B-cells (n=28)** | **Normal GC B-cells (n=1)** |
| --- | --- | --- | --- |
|  | **Median (range)** | **Median (range)** | **Median** |
| **Hypomethylated CpGs %** | 24.13 (15.82-47.52) | 26.57 (24.92-27.40) | 28.62 |
| **Semimethylated CpGs %** | 46.18 (24.92-60.80) | 19.99 (14.79-30.44) | 26.87 |
| **Hypermethylated CpGs %** | 28.73 (12.35-43.78) | 53.36 (44.65-57.82) | 44.52 |

Median (and range) within each methylation group, stated as percent of the total number of CpGs (n=670 233), for LBCL cases, normal B-cells, and normal GC B-cells. The statistical analysis was performed with the Chi-squared test and there was a significant difference in median CpG percentage between the three groups (χ^2^=19.696, df=4, p<0.001).

Table S2.
Hypo-, semi-, and hypermethylated CpGs based on the mean β-value of each CpG.

|  | **LBCL (n=93)** | **Normal B-cells (n=28)** | **Normal GC B-cells (n=1)** |
| --- | --- | --- | --- |
|  | **Total number (percent)** | **Total number (percent)** | **Total number (percent)** |
| **Hypomethylated CpGs %** | 143 101 (21.35%) | 176 728 (26.37%) | 191 788 (28.62%) |
| **Semimethylated CpGs %** | 384 689 (57.40%) | 140 086 (20.90%) | 180 071 (26.87%) |
| **Hypermethylated CpGs %** | 142 443 (21.25%) | 353 419 (52.73%) | 298 374 (44.52%) |

The number (and percent) of CpGs that were classified as hypo- (β<0.15), semi- (0.15≤β≤0.80), and hypermethylated (>0.80) in LBCL cases, normal B-cells, and normal GC B-cells based on the mean β-value of each CpG. The statistical analysis was performed with the Chi-squared test and there was a significant difference in median percentage between the three groups (χ^2^=36.46, df=4, p<0.001) or between LBCL and the normal GC B-cells (χ^2^=20.351, df=2, p<0.001).

Table S3.
Annotation distribution in LBCL cases and in normal CG B-cells.

|  | **Hypomethylated CpGs %** | | | **Semimethylated CpGs %** | | | **Hypermethylated CpGs %** | | |
| --- | --- | --- | --- | --- | --- | --- | --- | --- | --- |
|  | *LBCL* | *Normal GC B-cells* | *Adjusted p-value* | *LBCL* | *Normal GC B-cells* | *Adjusted p-value* | *LBCL* | *Normal GC B-cells* | *Adjusted p-value* |
| Northern Shelf | 1 | 1 | 1 | 4 | 4 | 1 | 5 | 5 | 1 |
| Northern shore | 13 | 13 | 1 | 10 | 12 | 1 | 8 | 8 | 1 |
| Island | 57 | 54 | 1 | 12 | 11 | 1 | 7 | 5 | 1 |
| Southern Shore | 12 | 11 | 1 | 8 | 9 | 1 | 7 | 7 | 1 |
| Southern Shelf | 1 | 1 | 1 | 4 | 3 | 1 | 5 | 5 | 1 |
| Open Sea | 16 | 20 | 1 | 63 | 61 | 1 | 68 | 72 | 1 |
| TSS1500 | 22 | 22 | 1 | 11 | 12 | 1 | 8 | 9 | 1 |
| TSS200 | 24 | 22 | 1 | 5 | 5 | 1 | 2 | 2 | 1 |
| 5’UTR | 19 | 18 | 1 | 10 | 10 | 1 | 8 | 8 | 1 |
| 1stExon | 3 | 3 | 1 | 1 | 1 | 1 | 1 | 1 | 1 |
| Body | 20 | 22 | 1 | 40 | 39 | 1 | 56 | 50 | 1 |
| 3’UTR | 1 | 1 | 1 | 3 | 2 | 1 | 4 | 4 | 1 |
| Intergenic | 11 | 13 | 1 | 30 | 31 | 1 | 22 | 26 | 1 |

The statistical analysis was performed with Fisher’s exact test and the p-values were adjusted for multiple testing. Percent of the total number of annotations within each methylation group were stated.

Table S4.
Epigenetic age in LBCL entities.

|  | **DLBCL-GC**  **(n=36)** | **DLBCL-nonGC**  **(n =30)** | **HGBL**  **(n=7)** | **PCNSL**  **(n=8)** | **t-DLBCL**  **(n=12)** | **p-value** |
| --- | --- | --- | --- | --- | --- | --- |
| **Age**  **(chronological years)** | 65.5 + 20.25 | 69.0 + 11.50 | 70.0 + 7.50 | 66.0 + 16.75 | 70.0 + 18.25 | 0.948 |
| **ΔHannum**  **(biological years)** | 15.6 + 27.6 | 15.5 + 21.1 | 24.2 + 27.4 | 28.1 + 12.2 | 14.0 + 24.5 | 0.434 |
| **ΔHorvath**  **(biological years)** | -0.9 + 21.9 | 2.4 + 28.1 | 4.3 + 13.1 | 23.2 + 27.5 | 6.4 + 25.8 | 0.184 |
| **ΔPhenoAge**  **(biological years)** | 12.8 + 29.7 | 18.2 + 26.5 | 6.2 + 29.9 | 58.3 +31.5 | 5.2 + 24.6 | **<0.001^a^** |
| **epiTOC**  **(mitotic age)** | 0.44 + 0.16 | 0.44 + 0.11 | 0.44 + 0.10 | 0.61 + 0.08 | 0.44 + 0.1 | **0.013^b^** |
| **MiAge**  **(mitotic age)** | 3546 + 2602 | 3408 + 1601 | 3697 + 1550 | 6295 + 2173 | 3708 + 1999 | **0.020^c^** |
| **epiCMIT**  **(mitotic age)** | 0.66 + 0.11 | 0.71 + 0.11 | 0.65 + 0.12 | 0.76 + 0.08 | 0.63 + 0.08 | 0.083 |
| **epiCMIT-hyper**  **(mitotic age)** | 0.55 + 0.16 | 0.58 + 0.11 | 0.52 + 0.13 | 0.74 + 0.11 | 0.56 + 0.14 | **0.042^d^** |
| **epiCMIT-hypo**  **(mitotic age)** | 0.66 + 0.11 | 0.71 + 0.11 | 0.65 + 0.12 | 0.76 + 0.08 | 0.63 + 0.07 | 0.080 |
| **DNAmTL**  **(kilobase)** | 6.89 + 0.45 | 7.07 + 0.51 | 6.70 + 0.44 | 7.29 + 0.39 | 6.94 + 0.48 | 0.072 |

^a^ PCNSL vs DLBCL-GC p=**0.002,** PCNSL vs DLBCL-nonGC p=**0.007**, PCNSL vs HGBL p=**0.009**, PCNSL vs t-DLBCL p<**0.001.**^b^ PCNSL vs DLBCL-GC p=**0.005,** PCNSL vs DLBCL-nonGC p=**0.018.** ^c^ PCNSL vs DLBCL-GC p=**0.017,** PCNSL vs DLBCL-nonGC p=**0.010.** ^d^ PCNSL vs DLBCL-GC p=**0.025.**

The statistical analysis was performed with the Kruskal-Wallis test (p-value) followed by ad hoc test with Bonferroni correction (significant p-values in footnotes). Significant p-values (p<0.05) are indicated as bold text. Median and IQR values were stated.

Table S5.
Standardized residuals of RTL in LBCL.

|  | **RTL_sres_ median and IQR** | **Total RTL_sres_ range** |
| --- | --- | --- |
| **DLBCL-GC (n=36)** | -0.669 + 1.929 | -4.673; 18.858 |
| **DLBCL-nonGC (n=30)** | -1.159 + 3.726 | -3.757; 6.824 |
| **HGBL (n=7)** | 1.483 + 2.160 | -2.038; 8.235 |
| **PCNSL (n=8)** | -2.041 + 4.324 | -3.772; 8.321 |
| **t-DLBCL (n=12)** | -1.222 + 1.811 | -2.298; 7.335 |

RTL_sres_ stated as median, IQR, and total range for each entity. The statistical analysis was performed with the Kruskal-Wallis test and there was no significant difference in RTL_sres_ between any of the entities (p=0.306).

Table S6.
Univariable survival analysis in DLBCL-GC and DLBCL-nonGC treated with R-CHOP-like regimens.

|  |  | **DSS** |  |  | **PFS** |  |
| --- | --- | --- | --- | --- | --- | --- |
| **Variables (n=56)** | **Events (n=12)** | **HR (95%CI)** | **P-value** | **Events (n=14)** | **HR (95%CI)** | **P-value** |
| **Age** |  |  |  |  |  |  |
| <median (n=31) | 7 | Reference |  | 9 | Reference |  |
| ≥median (n=25) | 5 | 1.042  (0.328-3.306) | 0.945 | 5 | 0.835  (0.272-2.563) | 0.752 |
| **aaIPI** |  |  |  |  |  |  |
| 0-1 (n=33) | 3 | Reference |  | 4 | Reference |  |
| 2-3 (n=23) | 9 | 5.126  (1.383-19.004) | **0.015** | 10 | 5.636  (1.548-20.520) | **0.009** |
| **Entity** |  |  |  |  |  |  |
| GC (n=31) | 5 | Reference |  | 5 | Reference |  |
| nonGC (n=25) | 7 | 1.869  (0.593-5.892) | 0.286 | 9 | 2.615  (0.875-7.816) | 0.085 |
| **RTL_sres_** |  |  |  |  |  |  |
| Normal (n=39) | 4 | Reference |  | 5 | Reference |  |
| Short (n=8) | 5 | 7.209  (1.921-27.050) | **0.003** | 6 | 6.513  (1.910-22.212) | **0.003** |
| Long (n=9) | 3 | 3.075  (0.679-13.913) | 0.145 | 3 | 2.464  (0.584-10.397) | 0.220 |
| **Hypomethylated CpGs %** |  |  |  |  |  |  |
| Q1-Q3 (n=33) | 4 | Reference |  | 6 | Reference |  |
| <Q1 (n=12) | 2 | 1.910  (0.346-10.552) | 0.458 | 2 | 1.206  (0.241-6.038) | 0.819 |
| >Q3 (n=11) | 6 | 6.080  (1.691-21.856) | **0.006** | 6 | 4.061  (1.291-12.775) | **0.017** |
| **Semimethylated CpGs %** |  |  |  |  |  |  |
| Q1-Q3 (n=26) | 4 | Reference |  | 4 | Reference |  |
| <Q1 (n=14) | 5 | 2.850  (0.760-10.679) | 0.120 | 5 | 3.178  (0.846-11.942) | 0.087 |
| >Q3 (n=16) | 3 | 1.039  (0.232-4.649) | 0.960 | 5 | 1.700  (0.448-6.444) | 0.435 |
| **Hypermethylated CpGs %** |  |  |  |  |  |  |
| >Q3 (n=15) | 2 | Reference |  | 2 | Reference |  |
| <Q1-Q3 (n=25) | 5 | 1.446  (0.278-7.522) | 0.661 | 5 | 1.507 (0.291-7.820) | 0.625 |
| <Q1 (n=16) | 5 | 1.990  (0.383-10.337) | 0.413 | 7 | 2.898  (0.590-14.230) | 0.190 |
| **Mean β-value** |  |  |  |  |  |  |
| >Q3 (n=14) | 1 | Reference |  | 1 | Reference |  |
| Q1-Q3 (n=30) | 7 | 2.795  (0.342-22.848) | 0.338 | 9 | 3.975 (0.502-31.492) | 0.191 |
| <Q1 (n=12) | 4 | 4.316  (0.479-38.874) | 0.192 | 4 | 4.245  (0.460-39.184) | 0.202 |
| **Global MVS** |  |  |  |  |  |  |
| <median (n=29) | 4 | Reference |  | 4 | Reference |  |
| ≥median (n=27) | 8 | 1.839  (0.546-6.199) | 0.326 | 10 | 2.367  (0.726-7.717) | 0.153 |
| **Promoter MVS** |  |  |  |  |  |  |
| <median (n=31) | 6 | Reference |  | 7 | Reference |  |
| ≥median (n=25) | 6 | 1.143  (0.368-3.551) | 0.817 | 7 | 1.153  (0.401-3.311) | 0.792 |
| **epiTOC (mitotic age)** |  |  |  |  |  |  |
| <median (n=31) | 5 | Reference |  | 7 | Reference |  |
| ≥median (n=25) | 7 | 1.811  (0.574-5.710) | 0.311 | 7 | 1.300  (0.455-3.714) | 0.625 |
| **CIMP** |  |  |  |  |  |  |
| Negative (n=13) | 1 | Reference |  | 1 | Reference |  |
| Positive (n=43) | 11 | 3.372  (0.435-26.138) | 0.245 | 13 | 3.936  (0.512-30.281) | 0.188 |

Univariable Cox proportional hazard model of disease-specific survival (DSS) and progression-free survival (PFS) in DLBCL-GC and DLBCL-nonGC treated with R-CHOP-like regimens. Quartile- and median classification were based on the entire LBCL cohort. Significant p-values (p<0.05, Wald’s test) are indicated as bold text.

Table S7.
Univariable survival analysis in LBCL treated with R-CHOP-like regimens.

|  |  | **DSS** |  |  | **PFS** |  |
| --- | --- | --- | --- | --- | --- | --- |
| **Variables (n=68)** | **Events (n=16)** | **HR (95%CI)** | **P-value** | **Events (n=20)** | **HR (95%CI)** | **P-value** |
| **Age** |  |  |  |  |  |  |
| <median (n=39) | 11 | Reference |  | 15 | Reference |  |
| ≥median (n=29) | 5 | 0.695  (0.240-2.008) | 0.501 | 5 | 0.505  (0.181-1.406) | 0.191 |
| **aaIPI** |  |  |  |  |  |  |
| 0-1 (n=42) | 5 | Reference |  | 8 | Reference |  |
| 2-3 (n=26) | 11 | 4.339  (1.503-12.527) | **0.007** | 12 | 3.441  (1.349-8.778) | **0.010** |
| **Entity** |  |  |  |  |  |  |
| GC (n=31) | 5 | Reference |  | 5 | Reference |  |
| nonGC (n=25) | 7 | 1.853  (0.588-5.842) | 0.292 | 9 | 2.605  (0.872-7.785) | 0.087 |
| HGBL (n=5) | 3 | 4.686  (1.109-19.798) | **0.036** | 3 | 5.407  (1.262-23.176) | **0.023** |
| t-DLBCL (n=7) | 1 | 1.099  (0.127-9.521) | 0.931 | 3 | 3.898  (0.906-16.774) | 0.068 |
| **RTL_sres_** |  |  |  |  |  |  |
| Normal (n=50) | 7 | Reference |  | 10 | Reference |  |
| Short (n=8) | 5 | 5.403  (1.700-17.176) | **0.004** | 6 | 4.250  (1.480-12.201) | **0.007** |
| Long (n=10) | 4 | 2.963  (0.856-10.252) | 0.086 | 4 | 2.031  (0.631-6.536) | 0.235 |
| **Hypomethylated**  **CpGs %** |  |  |  |  |  |  |
| Q1-Q3 (n=39) | 5 | Reference |  | 8 | Reference |  |
| <Q1 (n=16) | 4 | 2.537  (0.675-9.530) | 0.168 | 5 | 1.979  (0.639-6.127) | 0.237 |
| >Q3 (n=13) | 7 | 6.039  (1.892-19.279) | **0.002** | 7 | 3.711  (1.331-10.345) | **0.012** |
| **Semimethylated**  **CpGs %** |  |  |  |  |  |  |
| Q1-Q3 (n=35) | 7 | Reference |  | 9 | Reference |  |
| <Q1 (n=15) | 6 | 2.627  (0.876-7.880) | 0.085 | 6 | 2.212  (0.782-6.254) | 0.134 |
| >Q3 (n=18) | 3 | 0.695  (0.179-2.694) | 0.599 | 5 | 0.873  (0.288-2.645) | 0.810 |
| **Hypermethylated**  **CpGs %** |  |  |  |  |  |  |
| >Q3 (n=19) | 3 | Reference |  | 4 | Reference |  |
| <Q1-Q3 (n=32) | 8 | 1.448  (0.381-5.505) | 0.587 | 9 | 1.211  (0.370-3.960) | 0.752 |
| <Q1 (n=17) | 5 | 1.493  (0.353-6.304) | 0.586 | 7 | 1.555  (0.445-5.432) | 0.489 |
| **Mean β-value** |  |  |  |  |  |  |
| >Q3 (n=18) | 3 | Reference |  | 4 | Reference |  |
| Q1-Q3 (n=37) | 8 | 1.086  (0.286-4.118) | 0.904 | 11 | 1.141  (0.361-3.604) | 0.822 |
| <Q1 (n=13) | 5 | 2.218  (0.525-9.379) | 0.279 | 5 | 1.535  (0.396-5.950) | 0.535 |
| **Global MVS** |  |  |  |  |  |  |
| <median (n=39) | 7 | Reference |  | 9 | Reference |  |
| ≥median (n=29) | 9 | 1.518  (0.556-4.143) | 0.415 | 11 | 1.428  (0.577-3.535) | 0.441 |
| **Promoter MVS** |  |  |  |  |  |  |
| <median (n=39) | 8 | Reference |  | 11 | Reference |  |
| ≥median (n=29) | 8 | 1.300  (0.487-3.470) | 0.601 | 9 | 1.077  (0.443-2.616) | 0.870 |
| **epiTOC (mitotic age)** |  |  |  |  |  |  |
| <median (n=37) | 7 | Reference |  | 10 | Reference |  |
| ≥median (n=31) | 9 | 1.694  (0.630-4.552) | 0.296 | 10 | 1.320  (0.548-3.175) | 0.536 |
| **CIMP** |  |  |  |  |  |  |
| Negative (n=16) | 2 | Reference |  | 3 | Reference |  |
| Positive (n=52) | 14 | 2.223  (0.505-9.793) | 0.291 | 17 | 1.759  (0.512-6.040) | 0.369 |

Univariable Cox proportional hazard model of disease-specific survival (DSS) and progression-free survival (PFS) in LBCL treated with R-CHOP-like regimens. Quartile- and median classification were based on the entire LBCL cohort. Significant p-values (p<0.05, Wald’s test) are indicated as bold text.

Table S8.
Multivariable survival analysis in LBCL treated with R-CHOP-like regimens.

|  |  | **DSS** |  |  | **PFS** |  |
| --- | --- | --- | --- | --- | --- | --- |
| **Variables (n=68)** | **Events (n=16)** | **HR (95%CI)** | **P-value** | **Events (n=20)** | **HR (95%CI)** | **P-value** |
| **Age** |  |  |  |  |  |  |
| <median (n=39) | 11 | Reference |  | 15 | Reference |  |
| ≥median (n=29) | 5 | 0.942  (0.280-3.170) | 0.923 | 5 | 0.668  (0.220-2.027) | 0.476 |
| **aaIPI** |  |  |  |  |  |  |
| 0-1 (n=42) | 5 | Reference |  | 8 | Reference |  |
| 2-3 (n=26) | 11 | 5.079  (1.632-15.813) | **0.005** | 12 | 3.645  (1.340-9.910) | **0.011** |
| **Entity** |  |  |  |  |  |  |
| GC (n=31) | 5 | Reference |  | 5 | Reference |  |
| nonGC (n=25) | 7 | 1.997  (0.472-8.448) | 0.348 | 9 | 2.286  (0.610-8.567) | 0.220 |
| HGBL (n=5) | 3 | 17.427  (2.359-128.755) | **0.005** | 3 | 12.130  (2.028-72.545) | **0.006** |
| t-DLBCL (n=7) | 1 | 2.191  (0.202-23.830) | 0.519 | 3 | 6.190  (1.171-32.710) | **0.032** |
| **RTL_sres_** |  |  |  |  |  |  |
| Normal (n=50) | 7 | Reference |  | 10 | Reference |  |
| Short (n=8) | 5 | 6.011  (1.319-27.397) | **0.020** | 6 | 4.689  (1.102-19.963) | **0.037** |
| Long (n=10) | 4 | 2.731  (0.555-13.433) | 0.216 | 4 | 2.212  (0.506-9.674) | 0.292 |
| **Hypomethylated**  **CpGs %** |  |  |  |  |  |  |
| Q1-Q3 (n=39) | 5 | Reference |  | 8 | Reference |  |
| <Q1 (n=16) | 4 | 1.040  (0.211-5.122) | 0.962 | 5 | 1.029  (0.263-4.030) | 0.968 |
| >Q3 (n=13) | 7 | 5.147  (1.239-21.388) | **0.024** | 7 | 2.720  (0.784-9.435) | 0.115 |

Multivariable Cox proportional hazard model of disease-specific survival (DSS) and progression-free survival (PFS) in LBCL treated with R-CHOP-like regimens. Quartile- and median classification were based on the entire LBCL cohort. Significant p-values (p<0.05, Wald’s test) are indicated as bold text.


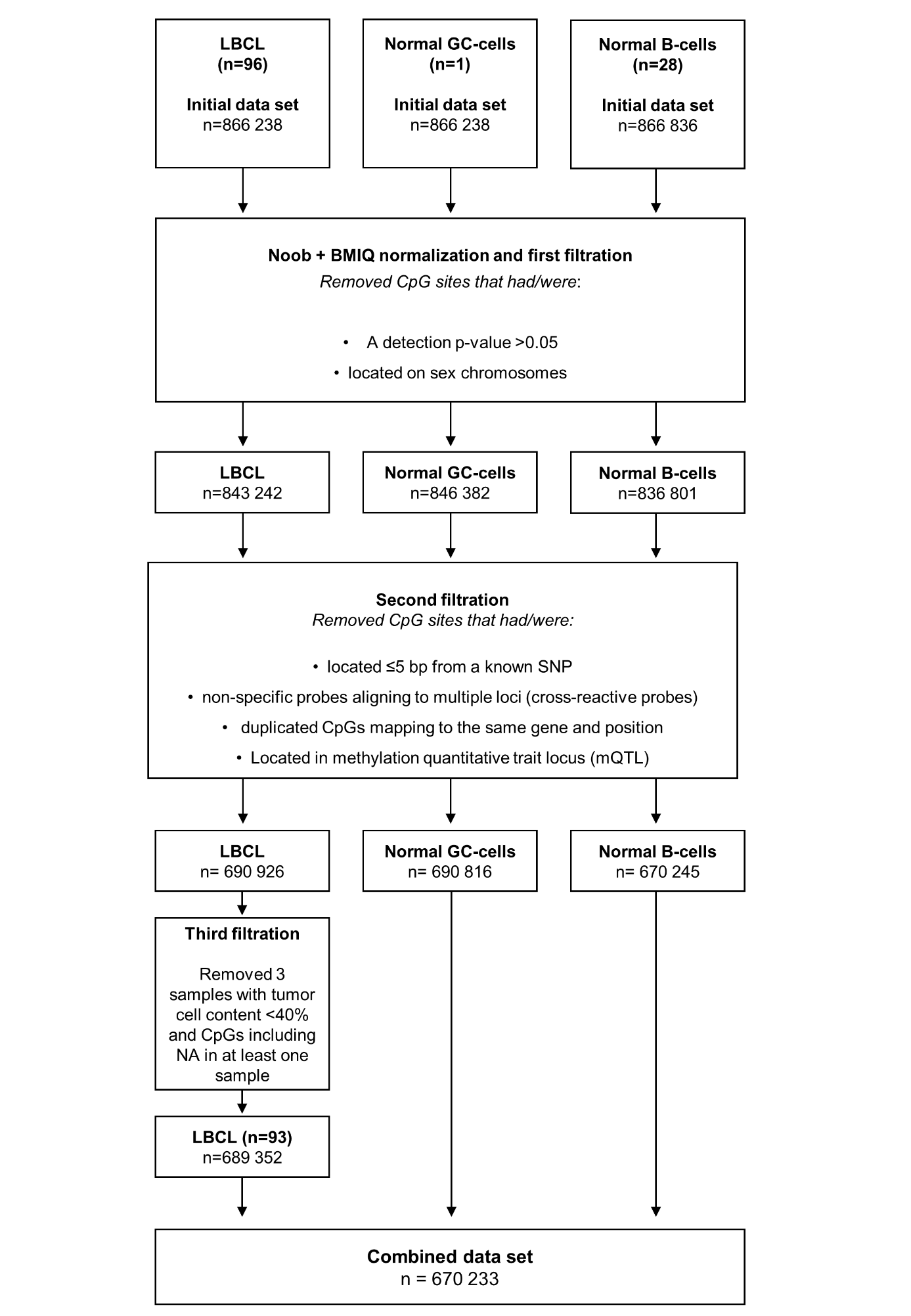


Figure S1.
Schematic overview of the normalization and filtration steps.

**
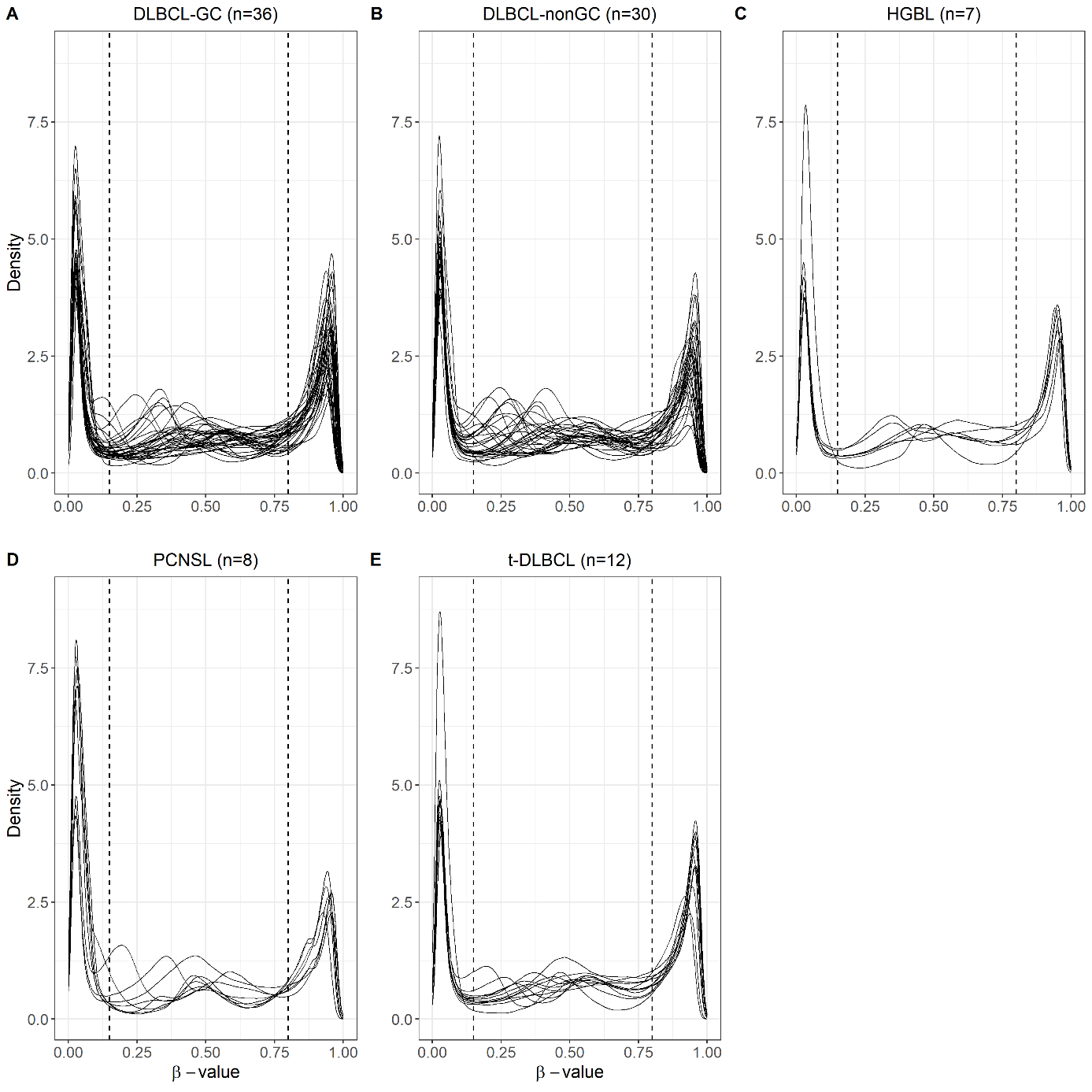
**

Figure S2.
Methylation distribution in LBCL entities. Density plots of β-value distribution in the LBCL data set (n=689 352 CpGs). The vertical dashed lines mark the cutoff for 0.15≤β≤0.8. A) DLBCL-GC (n=36), B) DLBCL-nonGC (n=30), C) HGBL (n=7), D) PCNSL (n=8), E) t-DLBCL (n=12).


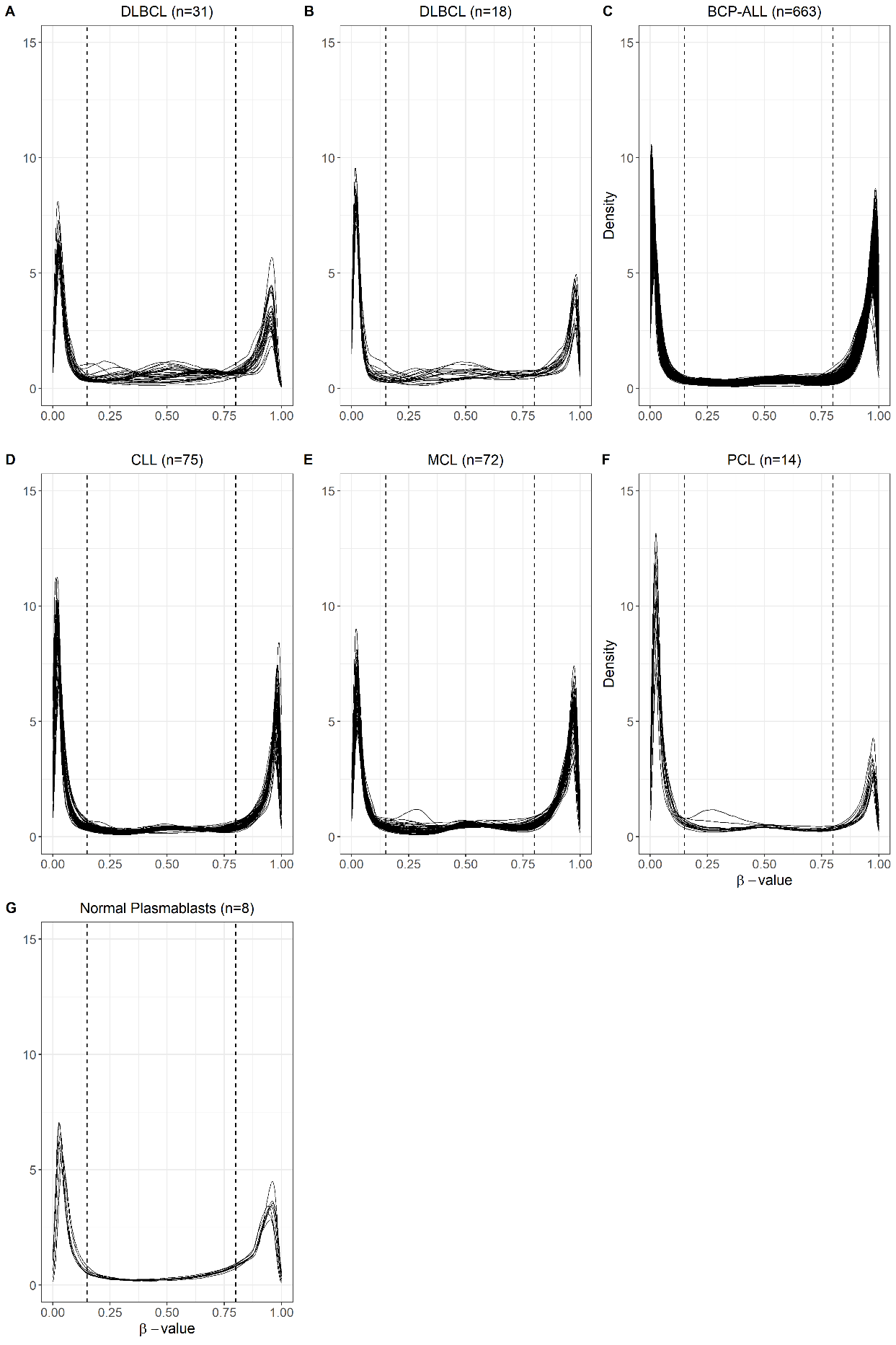


Figure S3.
Methylation distribution in B-cell neoplasms and normal cells**.** Density plots of β-value distribution. The vertical dashed lines mark the cutoff for 0.15≤β≤0.8. A) DLBCL (n=31, 341 726 CpGs), B) DLBCL (n=18, 340 565 CpGs), C) BCP-ALL (n=663, 339 043 CpGs), D) CLL (n=75, 342 362 CpGs), E) MCL (n=72, 687 224 CpGs), F) PCL (n=14, 344 170 CpGs), G) Normal plasmablasts (n=8, 344 295 CpGs).


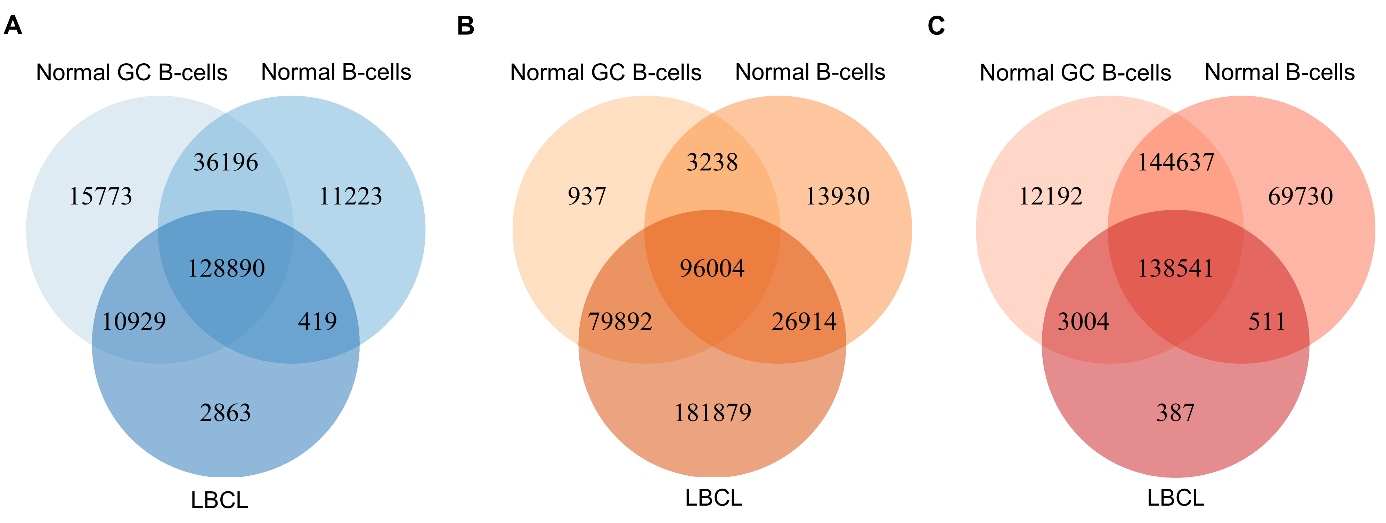


Figure S4.
Overlapping hypo-, semi-, and hypermethylated CpGs in the study cohort. The number of overlapping hypo-, semi-, and hypermethylated CpGs between LBCL, the normal GC B-cells, and the normal B-cells. The classification into methylation groups was based on the mean β-value of each CpG for cases and controls, respectively. A) Hypomethylated CpGs (blue), B) Semimethylated CpGs (orange), C) Hypermethylated CpGs (red).


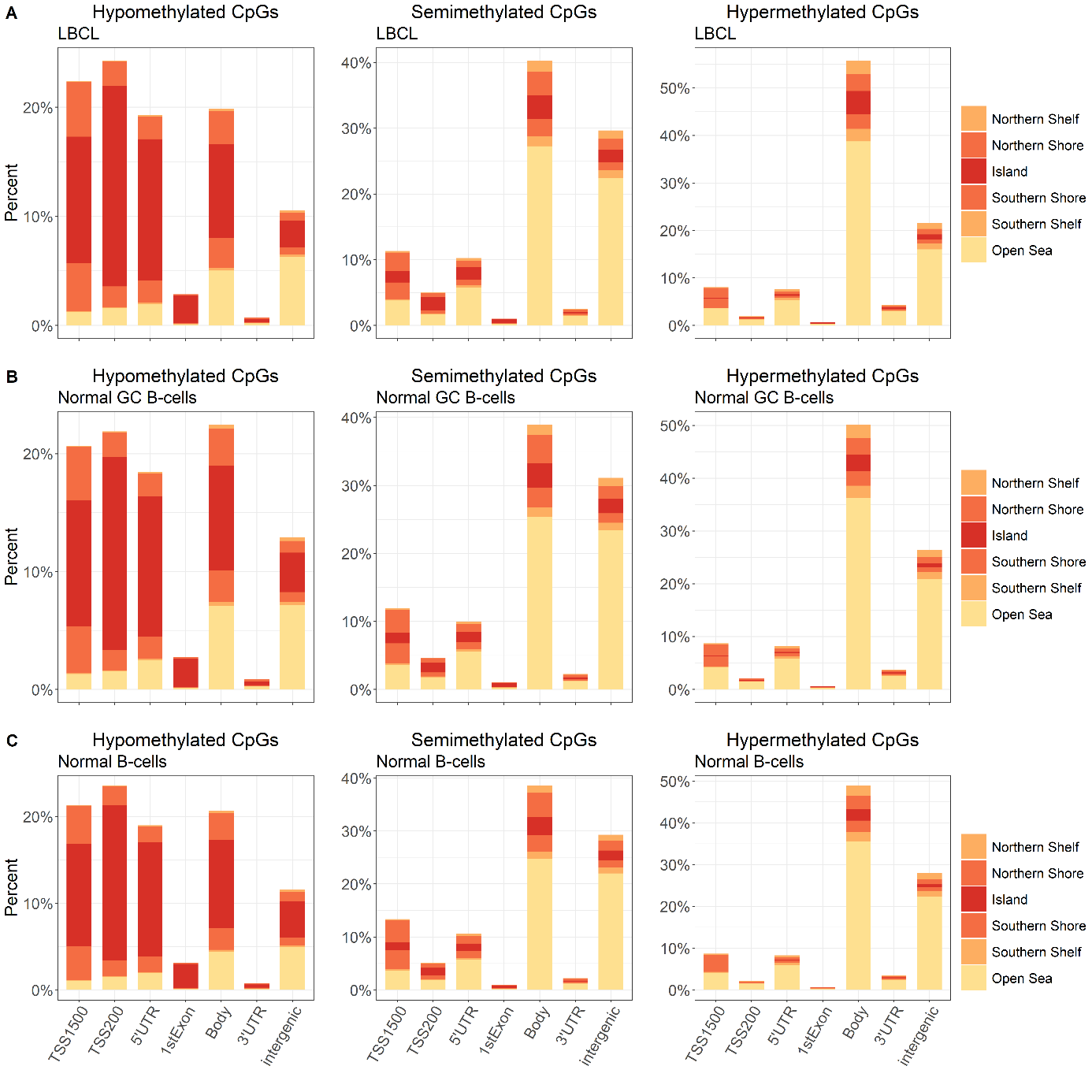


Figure S5.
Annotation distribution in LBCL cases, normal CG B-cells, and normal B-cells. Stacked barplot of the annotation distributions in the methylation groups in A) LBCL, B) Normal GC B-cells, C) Normal B-cells. The y-axis shows the percentage of the total number of annotations within each methylation group. The x-axis represents the genomic location. Colors represent the relation to CpG island. TSS1500/TSS200 = 1500-200/200-0 bases upstream of the transcription start site. 5’UTR/3’UTR = within the 5’/3’ untranslated region. Northern/southern shelf = 2-4 kb upstream/downstream from a CGI. Northern/southern shore = 0-2 kb upstream/downstream from a CGI. Open sea = more than 4 kb from a CGI.

**
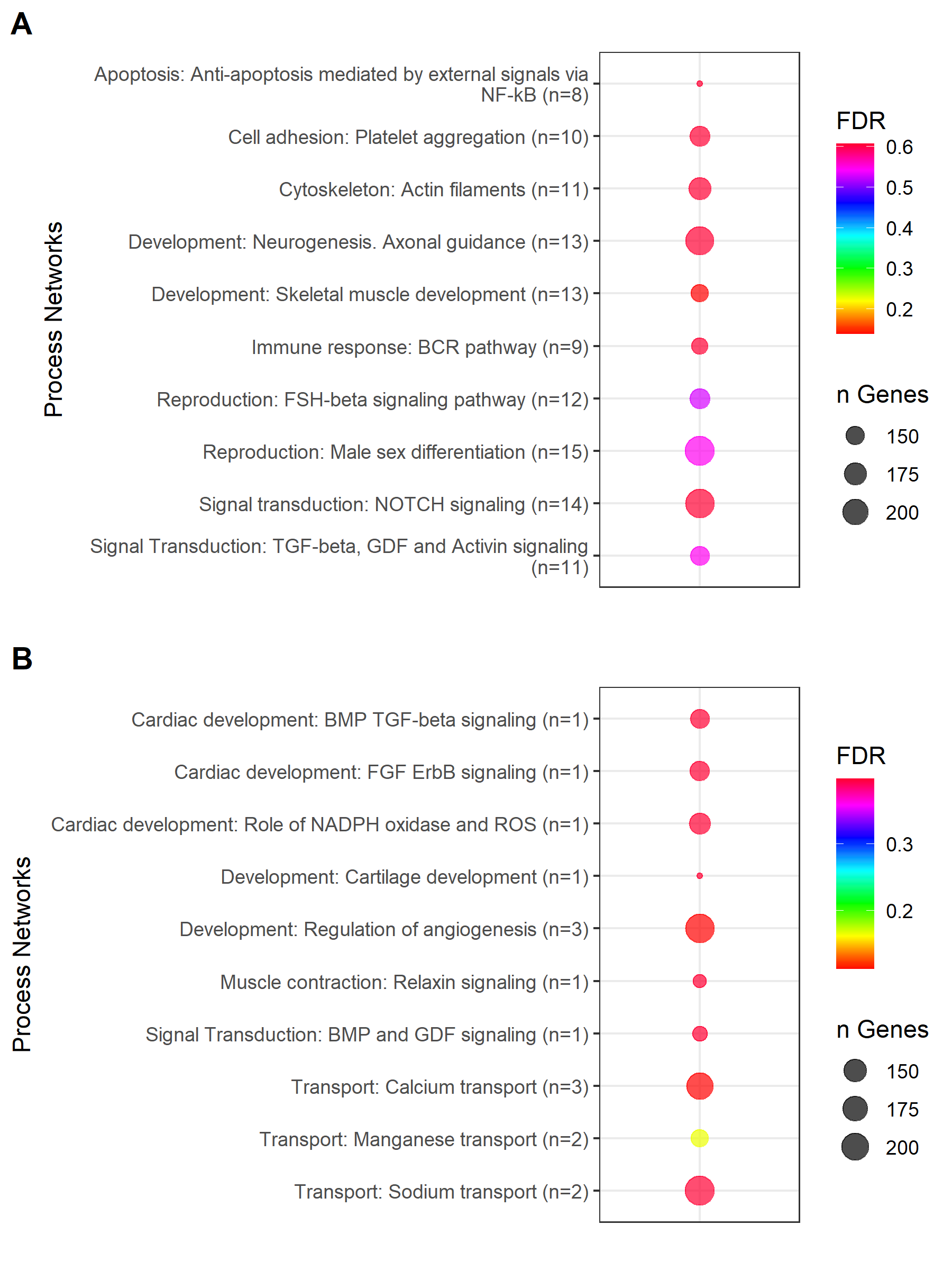
** Figure S6.
Networks associated with the genes that were differentially methylated between DLBCL-GC and DLBCL-nonGC. The figure includes the top ten process networks from the GeneGO MetaCore database analysis. Numbers in parentheses on the y-axis indicates the number of genes identified among the DM-CpGs that were involved in the network. The circles were annotated after color and size, where color corresponds to the false discovery rate (FDR) and the size to the total number of genes involved in the network (provided by GeneGO MetaCore). A) Networks associated with genes with lower mean methylation in DLBCL-nonGC compared to DLBCL-GC. B) Networks associated with genes with higher mean methylation in DLBCL-nonGC compared to DLBCL-GC.


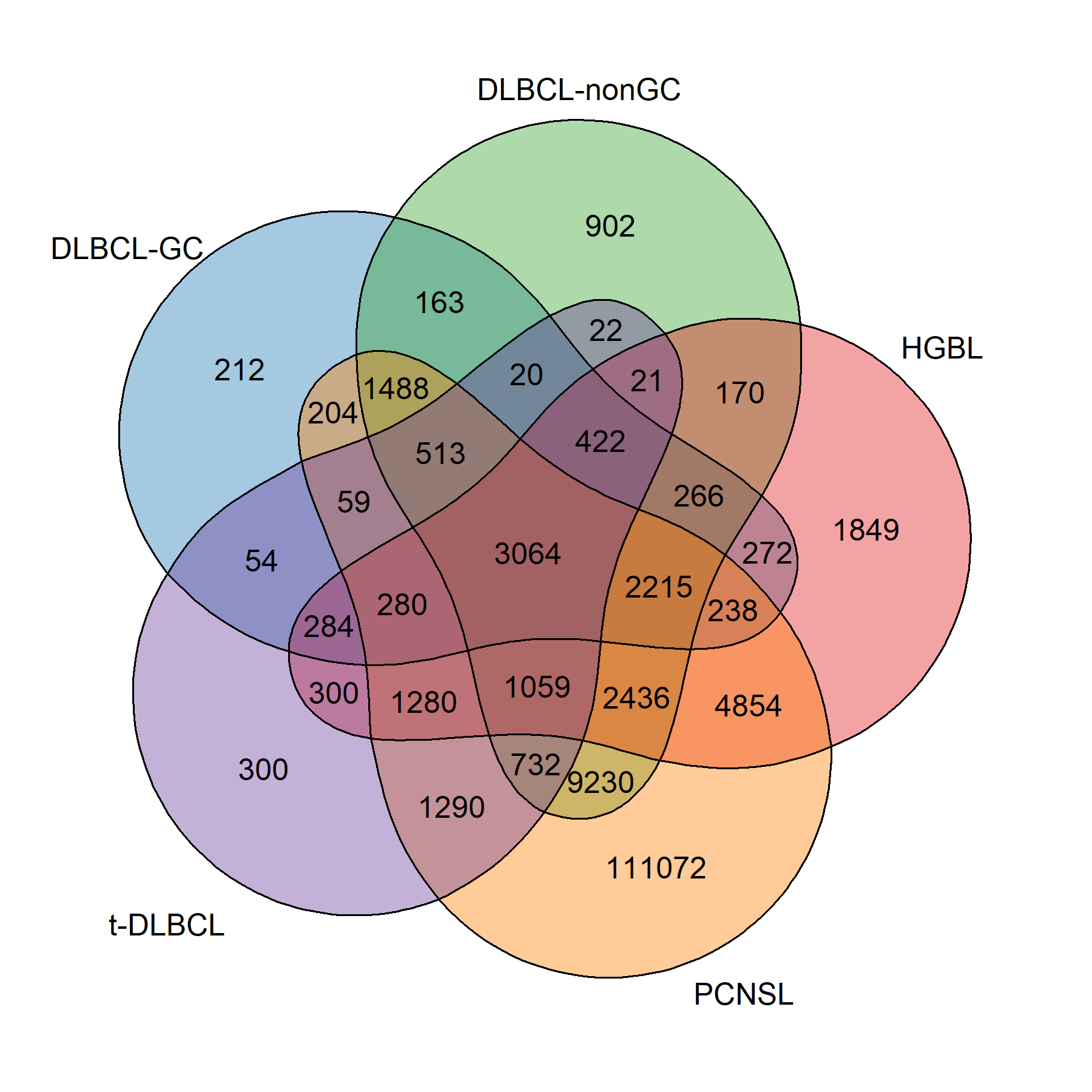


Figure S7.
Overlapping differentially methylated CpGs in LBCL entities compared to normal GC B-cells. The cutoff was mean |Δβ|≥0.4. In total, the number of DM-CpGs were: 9 754 (DLBCL-GC), 22 723 (DLBCL-nonGC), 19 010 (HGBL), 140 014 (PCNSL), and 9 700 (t-DLBCL). The number of DM-CpGs that were overlapping between all entities was 3064.

**
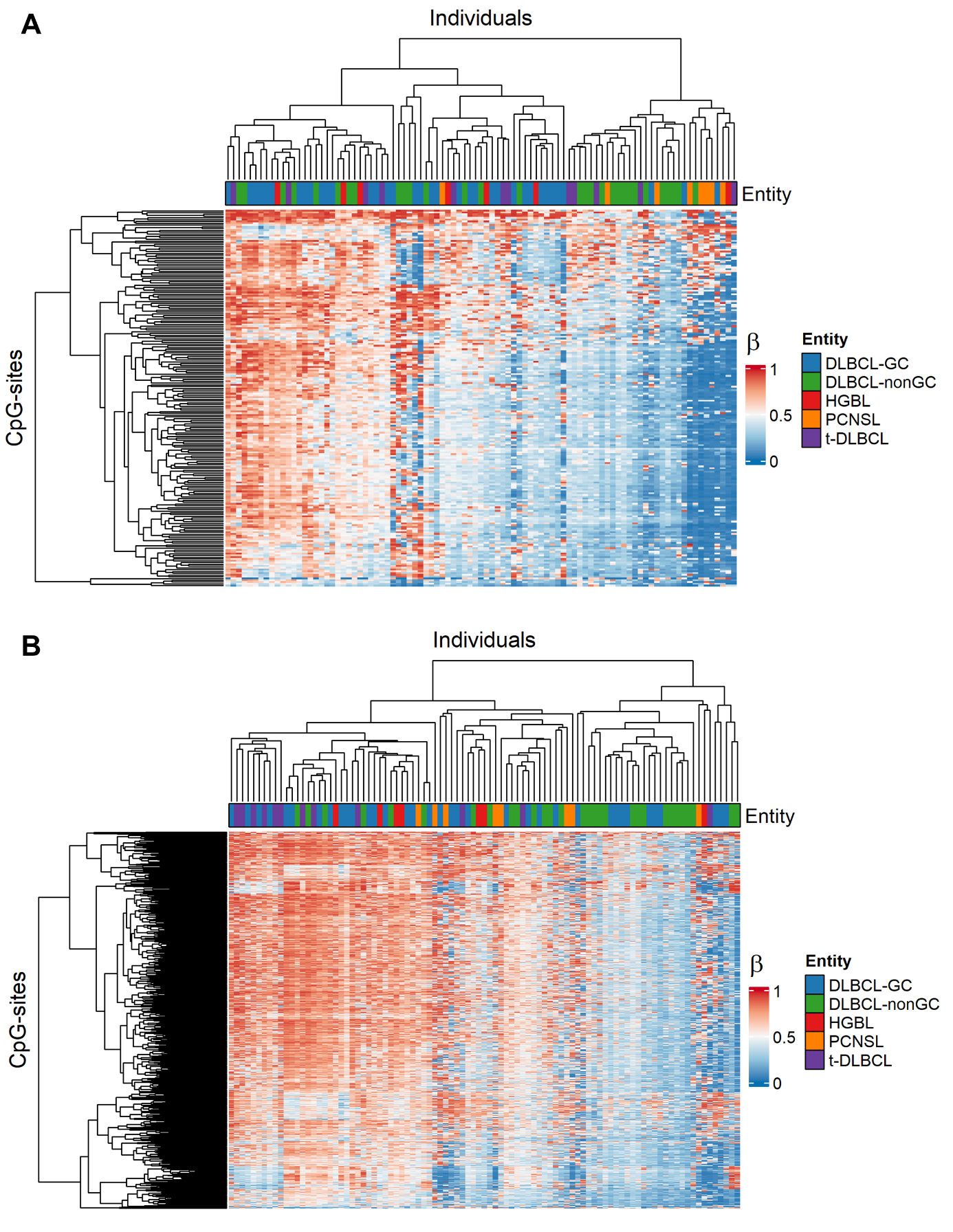
**

Figure S8.
Heatmap clustering of the unique DM-CpGs identified in DLBCL-GC and DLBCL-nonGC. Clustering of the unique DM-CpG sites (cutoff = mean |Δβ|≥0.4) in A) DLBCL-GC (n=212), B) DLBCL- nonGC (n=902). Rows correspond to CpGs, which are color-coded after methylation level: β=0 is blue and β=1 is red. Columns represent samples and the annotation bar below the column dendrogram is colored after entity.


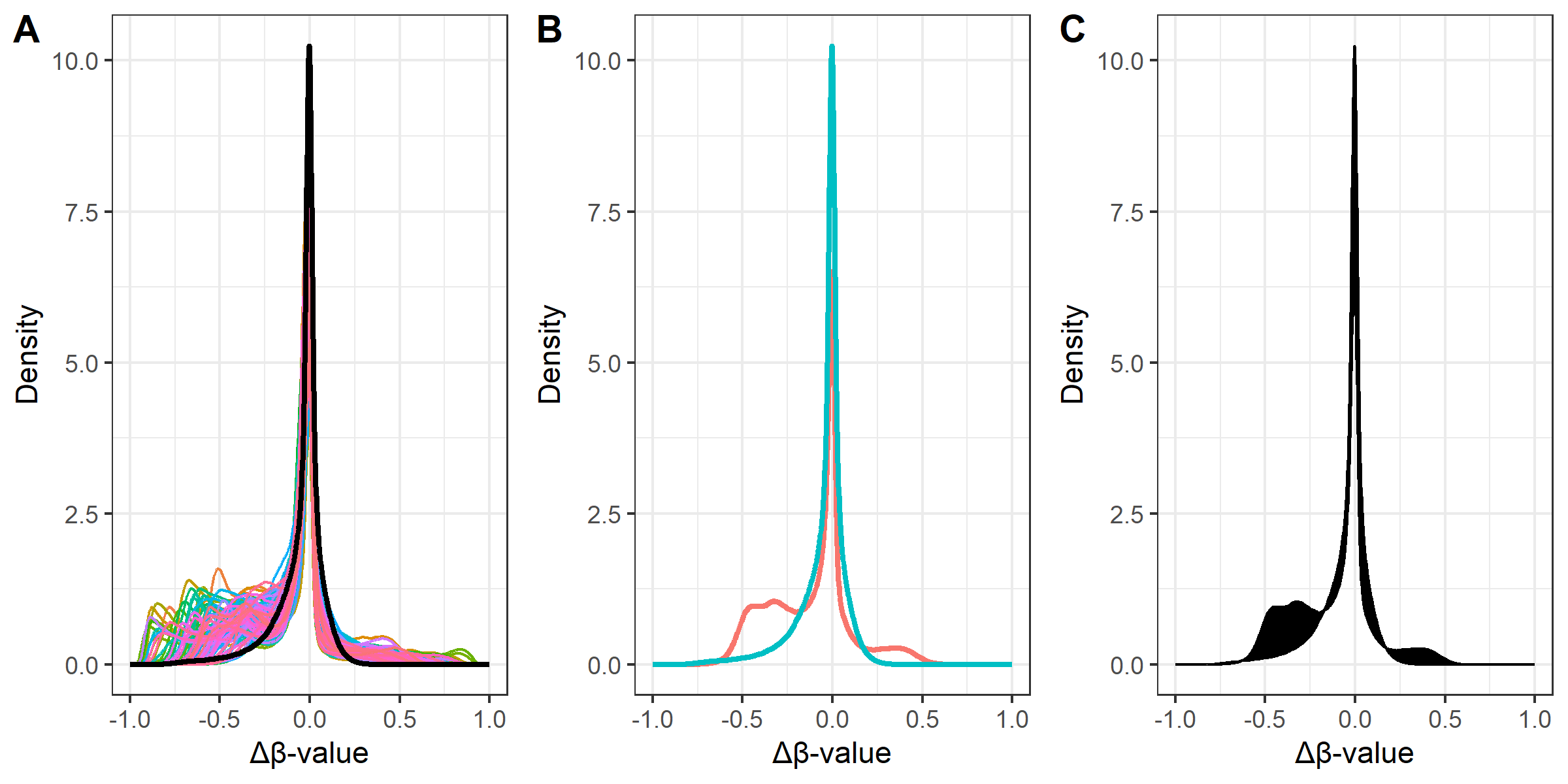


Figure S9.
Methylation variability score in LBCL cases. Density plots of A) The methylation difference (Δβ) between the normal B-cells and normal GC B-cells (black line, representing normal activation-specific methylation alterations) and the normal B-cells and each LBCL sample (colored lines). B) the methylation difference (Δβ) of normal B-cells compared to one LBCL sample (red line), and the normal GC B-cells (blue line). C) The non-overlapping region between one LBCL sample and the normal GC B-cells (black), which area corresponds to the MVS.


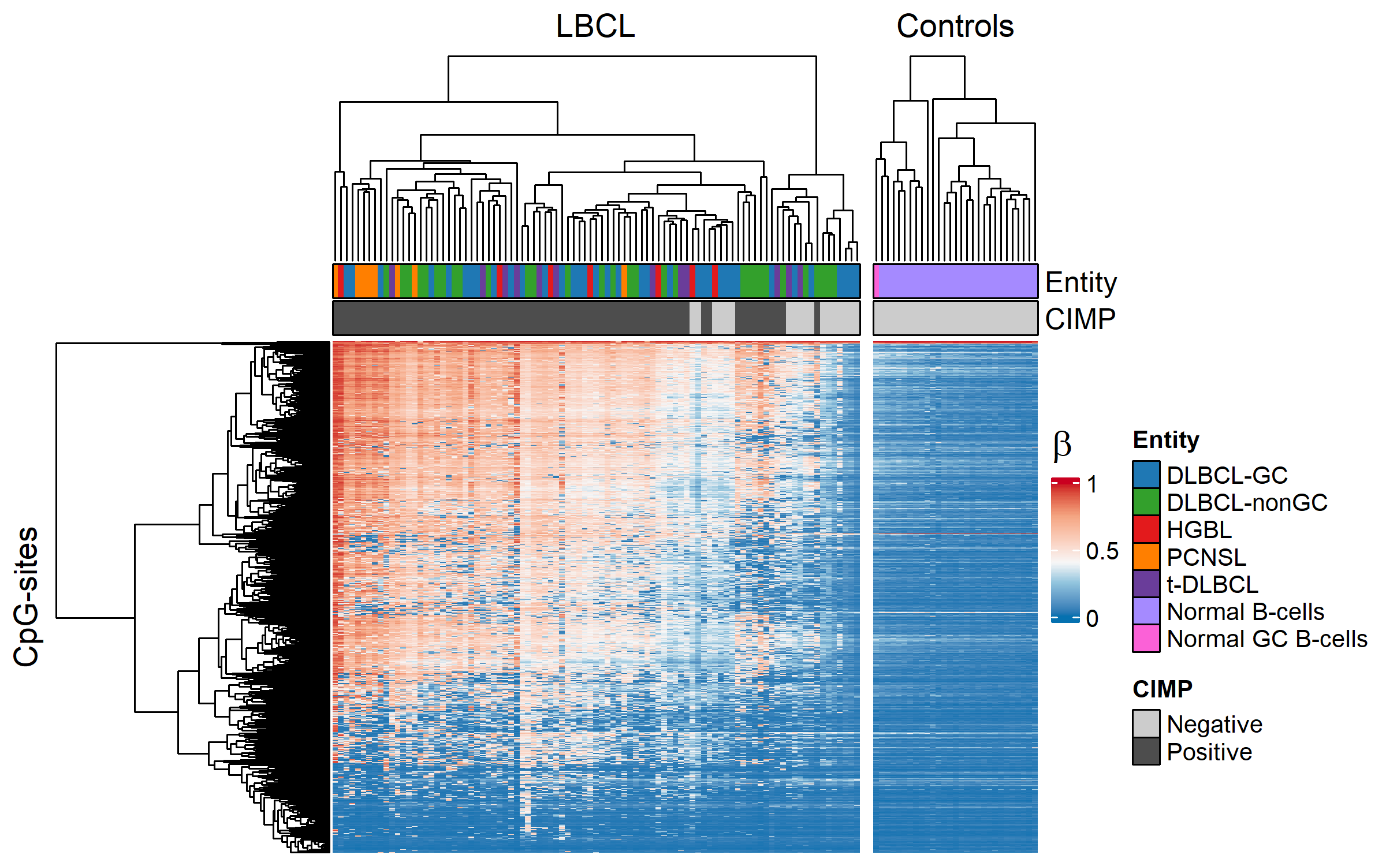


Figure S10.
CpG island Methylator Phenotype in the study cohort. Heatmap clustering of the 1091 CpG sites from the CIMP panel that were present in the combined LBCL, normal GC B-cell, and normal B-cell data sets. Rows correspond to CpGs, which are color-coded after methylation level: β=0 is blue and β=1 is red. Columns represent samples and the annotation bar below the column dendrogram is colored after entity and CIMP classification.

**
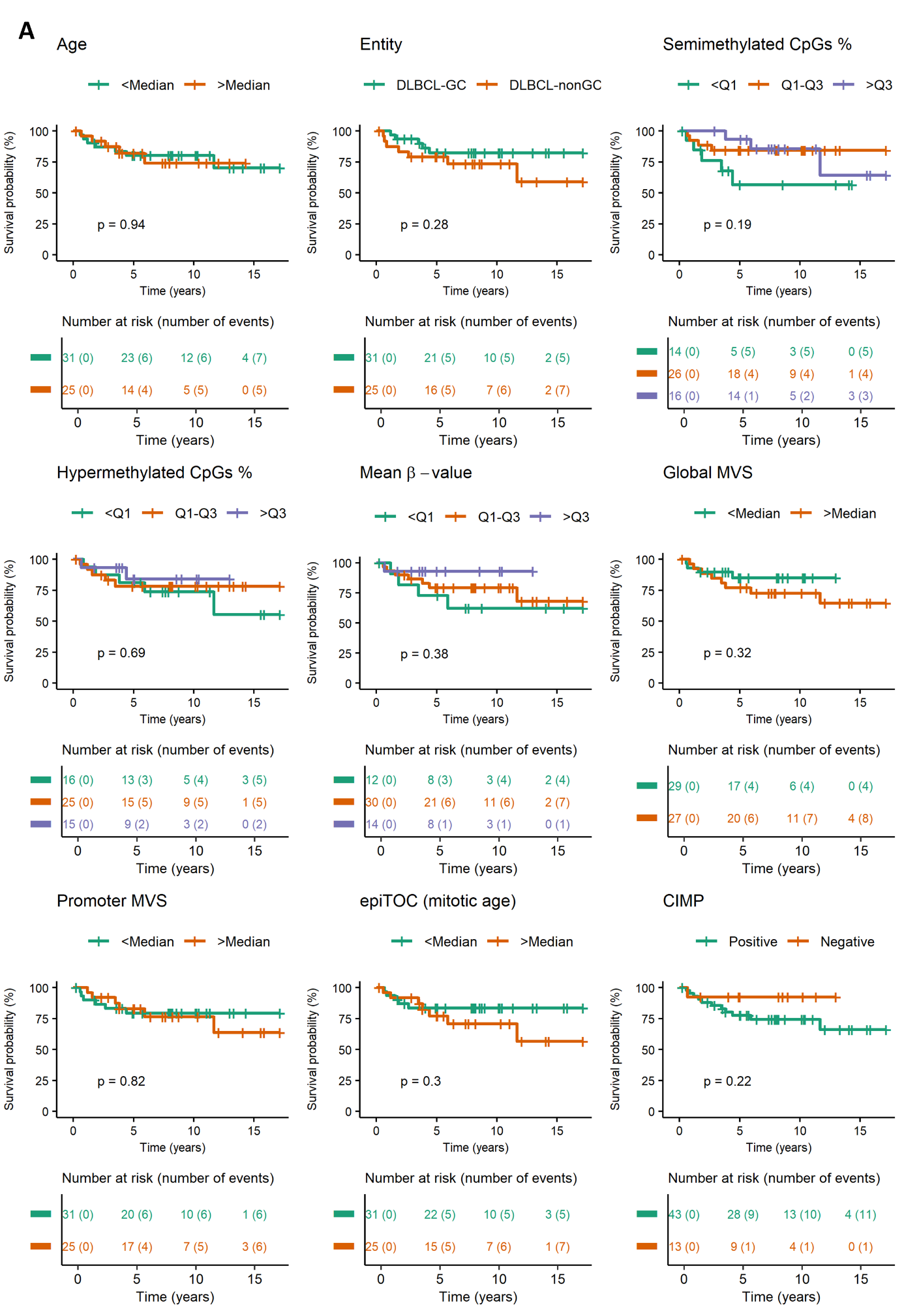
**
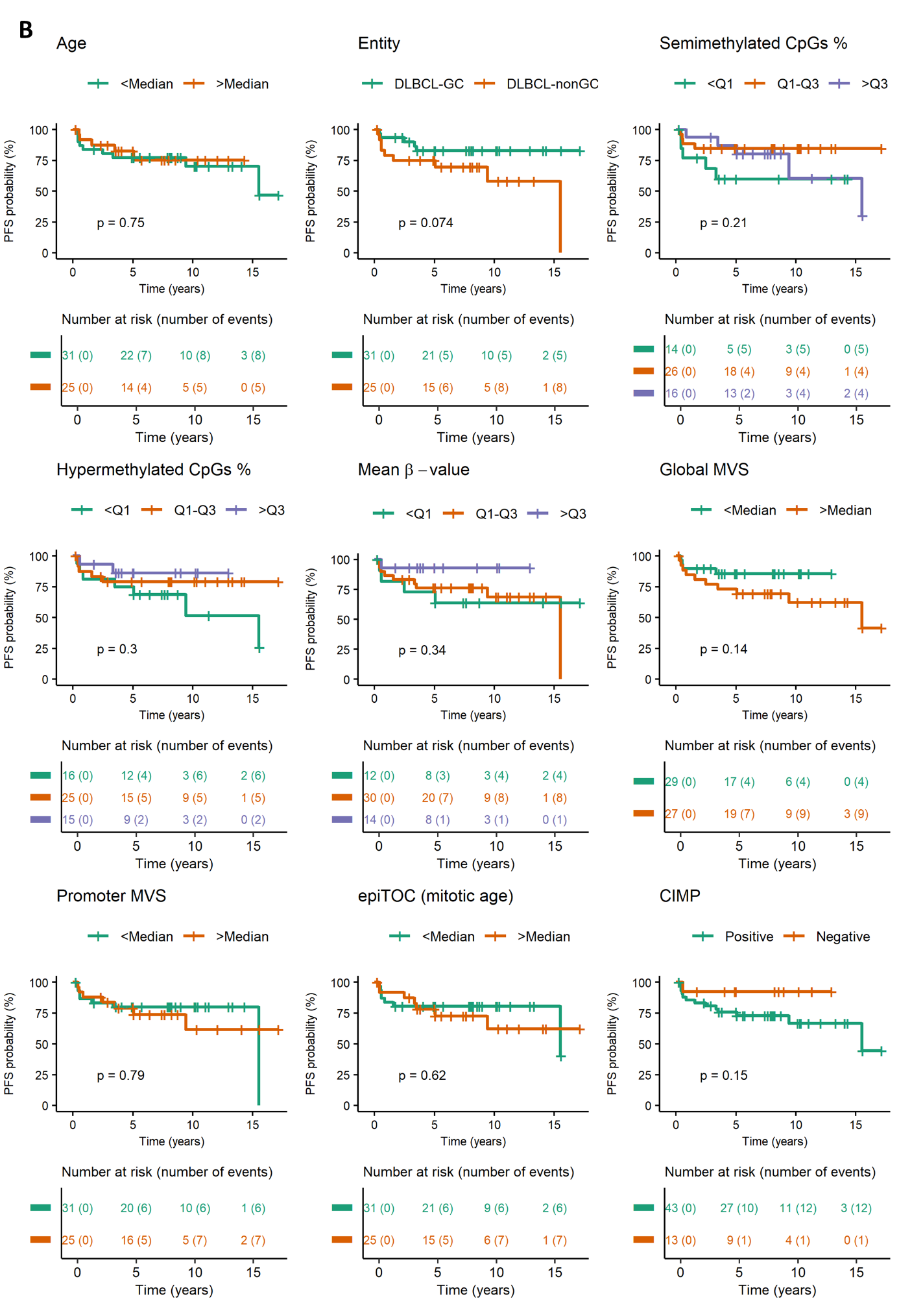


Figure S11.
Disease-specific survival and progression-free survival analysis. Non-significant Kaplan-Meier curves and risk tables of DLBCL-GC and DLBCL-nonGC cases treated with R-CHOP-like regimens (n=56). The cause of death was stated as death by lymphoma (n=12). Progression was stated as progression or relapse at any time during follow-up (n=14). p-values were retrieved from the Log-rank test. A) DSS, B) PFS.

**
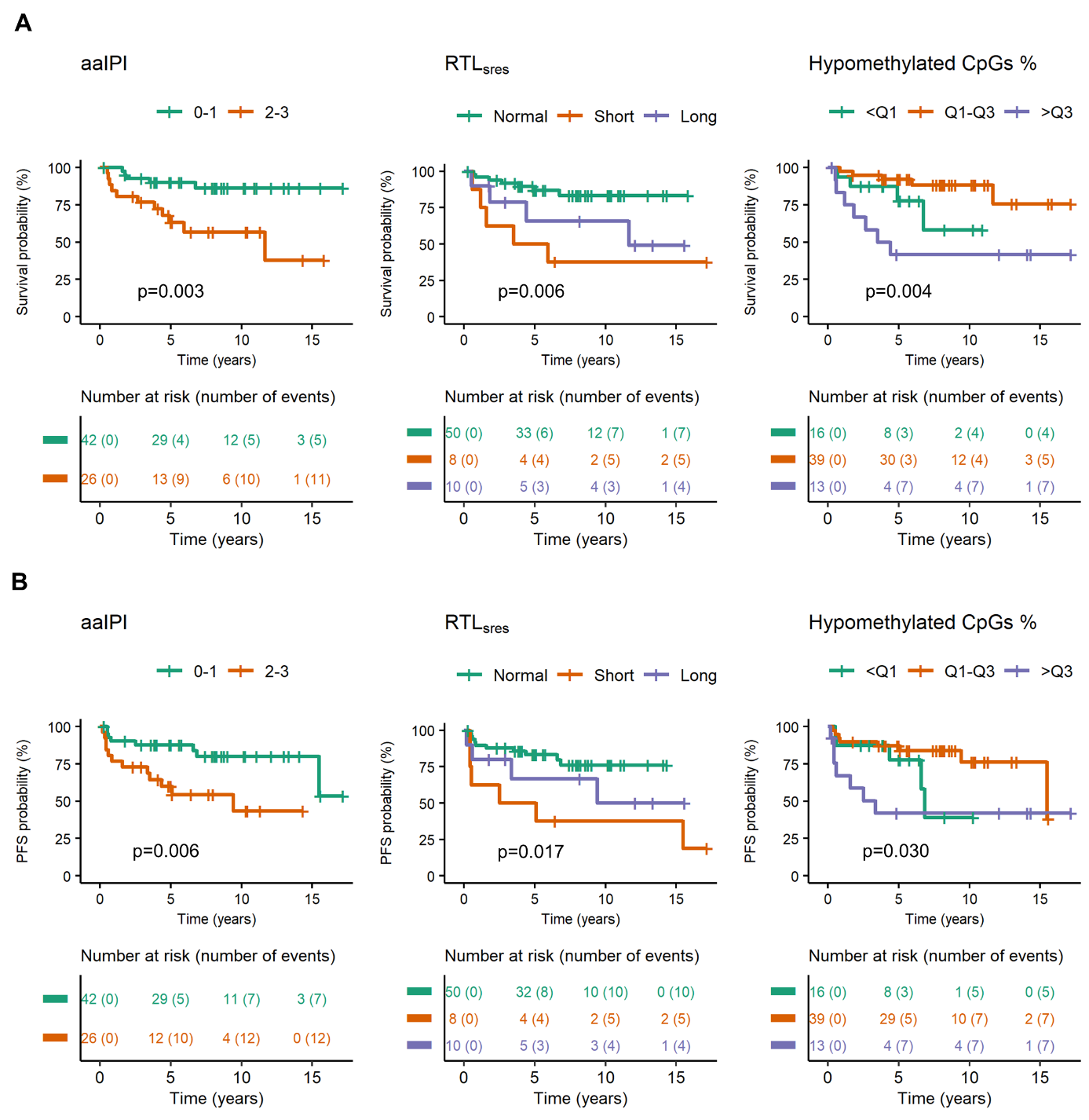
**

Figure S12.
Disease-specific survival and progression-free survival analysis. Significant Kaplan-Meier curves and risk tables of LBCL cases treated with R-CHOP-like regimens (n=68). The cause of death was stated as death by lymphoma (n=16). Progression was stated as progression or relapse at any time during follow-up (n=20). p-values were retrieved from the Log-rank test. A) DSS, B) PFS.

**
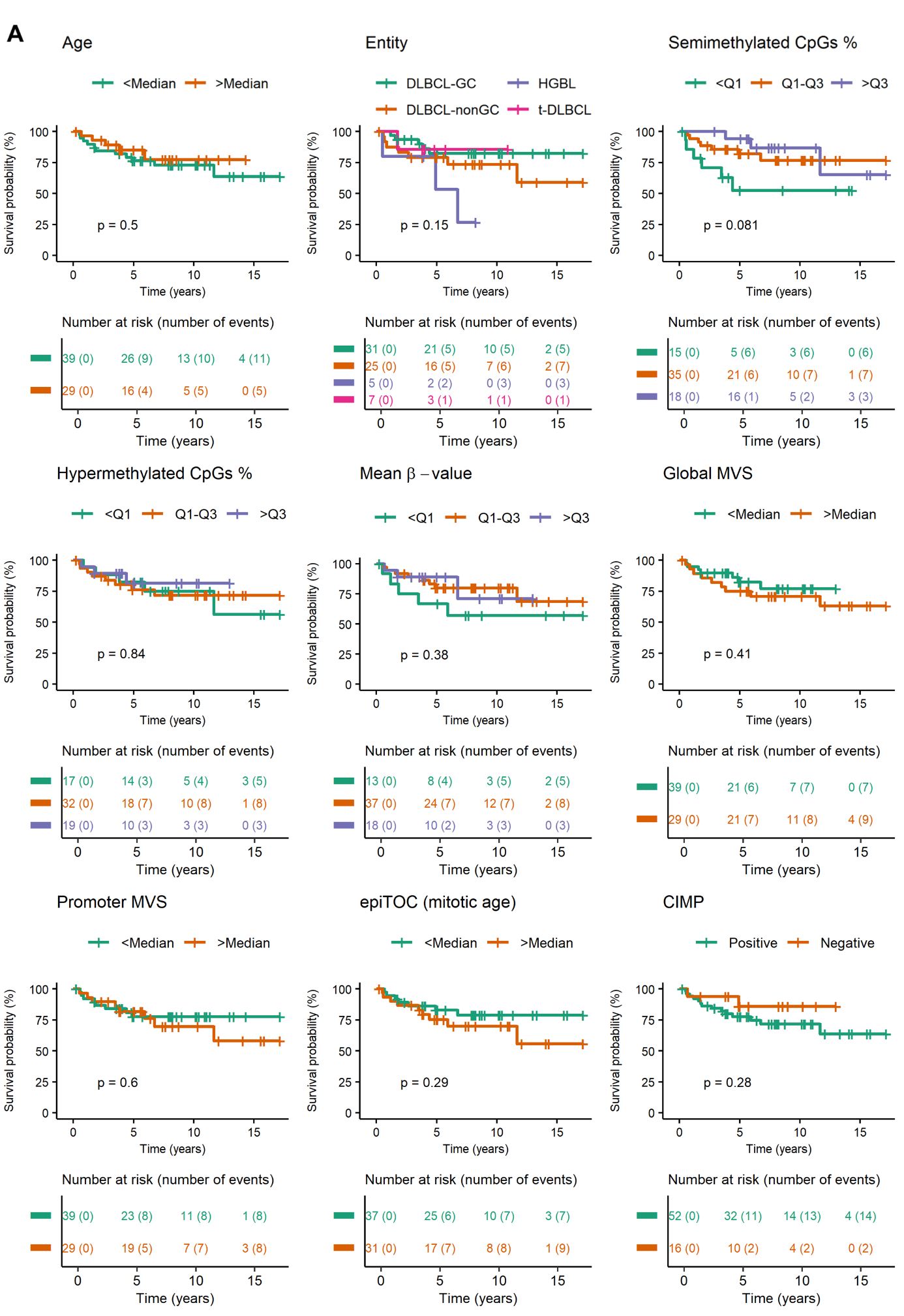

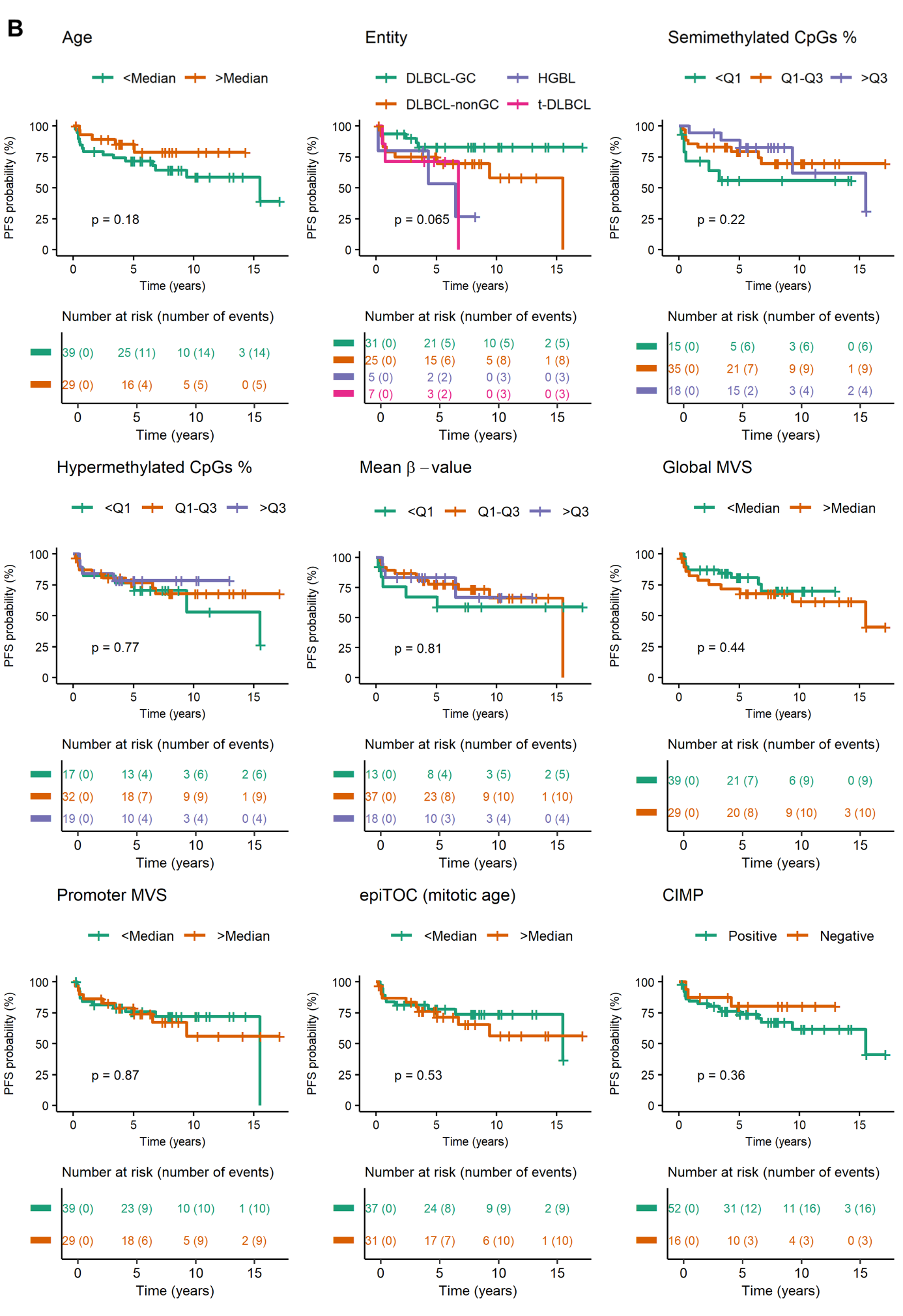
**

Figure S13.
Disease-specific survival and progression-free survival analysis. Non-significant Kaplan-Meier curves and risk tables of in LBCL cases treated with R-CHOP-like regimens (n=68). The cause of death was stated as death by lymphoma (n=16). Progression was stated as progression or relapse at any time during follow-up (n=20). p-values were retrieved from the Log-rank test. A) DSS, B) PFS.

**
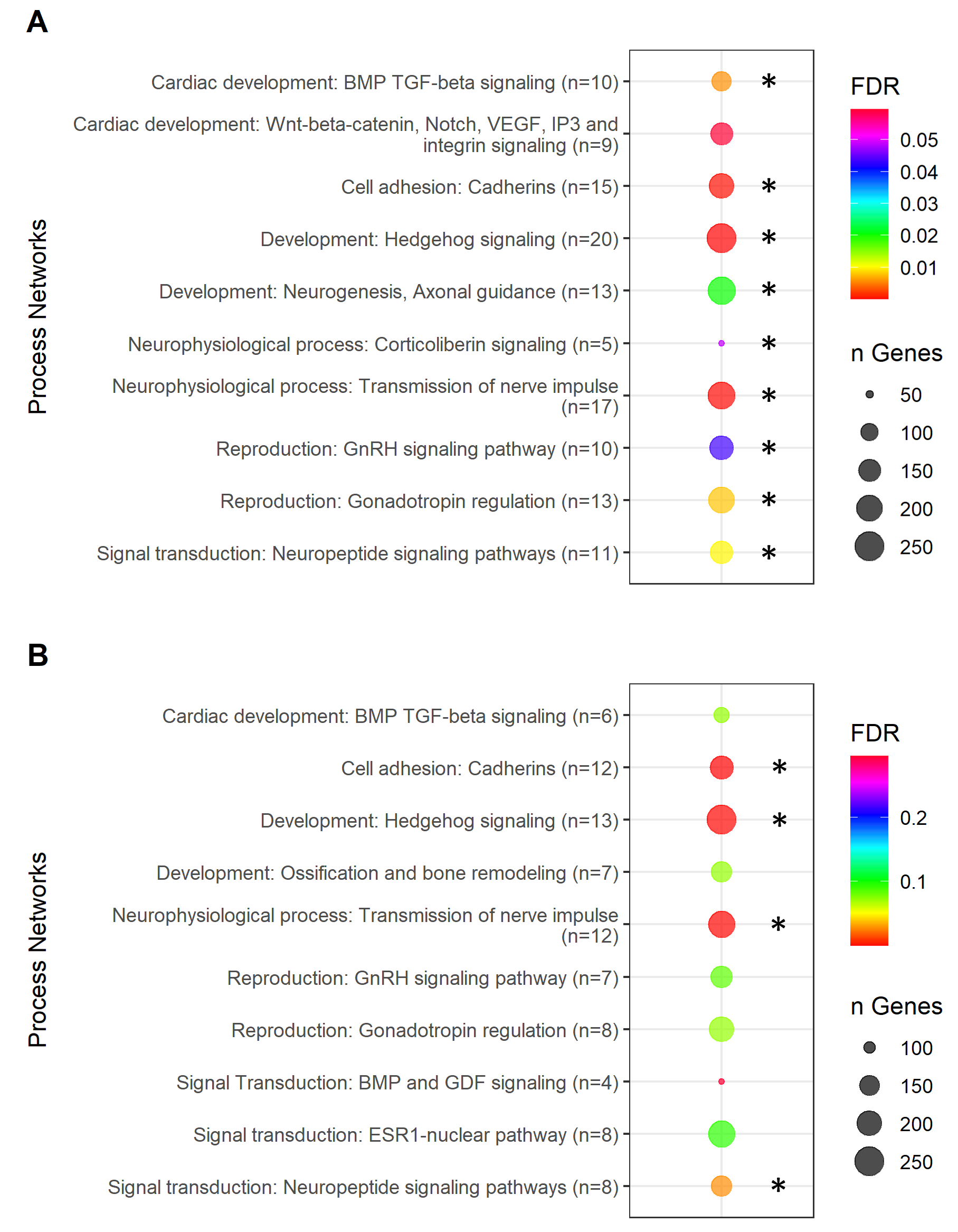
**

Figure S14.
Networks associated with the genes that had enriched CGI hypermethylation in the R-CHOP-like treated DLBCL cases. The Hypo>Q3 group was compared to the other R-CHOP-like treated DLBCL cases (Hypo<Q1-Q3). The figure includes the top ten process networks from the GeneGO MetaCore database analysis. Asterisk indicate significance. Numbers in parentheses on the y-axis indicates the number of genes identified in Hypo>Q3 that were involved in the network. The circles were annotated after color and size, where color corresponds to the false discovery rate (FDR) and the size to the total number of genes involved in the network (provided by GeneGO MetaCore). A) Networks associated with genes with enriched CGI hypermethylation in Hypo>Q3 compared to Hypo<Q1-Q3 B) Networks associated with genes with enriched promoter-associated CGI hypermethylation in Hypo>Q3 compared to Hypo<Q1-Q3.
